# Metabolomics insights into the protective molecular mechanism of *Vaccinium myrtillus* against oxidative stress in intestinal cells

**DOI:** 10.1038/s41598-025-93722-x

**Published:** 2025-03-13

**Authors:** Sara Novi, Vicky Caponigro, Maria Rosaria Miranda, Giovanna Aquino, Matteo Delli Carri, Emanuela Salviati, Silvia Franceschelli, Carla Sardo, Manuela Giovanna Basilicata, Vincenzo Vestuto, Mario Felice Tecce, Federico Marini, Giacomo Pepe, Pietro Campiglia, Michele Manfra

**Affiliations:** 1 Department of Pharmacy, University of Salerno, Via G. Paolo II, 84084 Fisciano, Salerno Italy; 2 Drug Discovery and Development, University of Salerno, 84084 Fisciano, Salerno Italy; 3 Department of Advanced Medical and Surgical Sciences, University of Campania “Luigi Vanvitelli”, Naples, Italy; 4 Department of Chemistry, University of Rome “La Sapienza”, Piazzale Aldo Moro 5, 00185 Rome, Italy; 5 National Biodiversity Future Center (NBFC), 90133 Palermo, Italy; 6 Department Health Science, University of Basilicata, Viale dell’Ateneo Lucano, 85100 Potenza, Italy

**Keywords:** High-resolution mass spectrometry, Metabolomics, Food science, Antioxidants, In-cell studies, Blueberry, Biochemistry, Biological techniques, Drug discovery, Chemistry

## Abstract

Blueberry (*Vaccinium myrtillus L.*) is a rich source of secondary metabolites known for their potent antioxidant, anti-inflammatory, and cytoprotective properties. These compounds are essential in neutralizing reactive oxygen species (ROS), which are implicated in oxidative stress-related diseases. In this study, we induced oxidative stress in IEC-6 small intestine cells using hydrogen peroxide (H_2_O_2_), creating a cellular model to investigate the biochemical response. The obtained results showed that a blueberry extract (BLUBE) significantly exhibited strong antioxidant capacity, as evidenced by DPPH, FRAP and ABTS in vitro tests. Additionally, BLUBE effectively inhibited the release of reactive species in cells and enhanced cytoprotective response, as indicated by improved wound healing and clonogenic potential reduction of stress fibers rearrangement and apoptosis. Metabolomic analysis, specifically High-Resolution Mass Spectrometry (HR-MS), was employed to elucidate the metabolic alterations associated with the protective activity of BLUBE against oxidative stress in IEC-6 cells. Chemometric approaches were applied to preprocess the data, explore variability, and identify systematic biases, ensuring the removal of batch effects and other experimental artifacts. A Partial Least Squares Discriminant Analysis classification model confirmed clear group stratifications with high accuracy (98.75 ± 2.31%), sensitivity, and specificity, aiding in the identification of significant metabolites for pathway enrichment analysis. Key metabolic pathways, including sphingolipid metabolism, taurine and hypotaurine metabolism, glycerophospholipid metabolism, and cysteine and methionine metabolism, were significantly modulated, supporting the biochemical basis of BLUBE’s protective effects. In fact, BLUBE was able to partially reverse the downregulation of these pathways, effectively reducing oxidative stress and promoting cell survival. This study highlights the power of HR-MS-based metabolomics in uncovering the mechanisms of nutraceuticals and emphasizes the potential of BLUBE as a protective agent for oxidative stress-related diseases. It also underscores the growing significance of metabolomics in the food and pharmaceutical industries.

**Supplementary Information:**

The online version contains supplementary material available at 10.1038/s41598-025-93722-x.

## Introduction

Recently, natural substances and nutraceuticals have attracted significant attention for their broad therapeutic effects and low toxicity, particularly in the management of chronic diseases^1–4^. In cancer prevention and treatment, nutraceuticals inhibit cancer cell proliferation and differentiation, block efflux transporters, and help reduce the toxicity of chemotherapy drugs, such as cardiotoxicity and hepatotoxicity^5–9^. Additionally, many natural compounds exhibit antioxidant properties, protecting against free radicals that damage DNA and cell membranes^10^. This antioxidant action plays a key role in preserving the integrity of epithelial barriers, like the intestinal lining, which, when compromised, allows ingested materials and pathogens to trigger inflammation and oxidative stress. The latter is now recognized as a potentially critical factor in the onset, progression, and severity of chronic inflammatory disorders^11–16^.

Blueberries (*Vaccinium myrtillus L.*) are among the fruits that are best recognized for their potential health benefits^17–19^. Many of their beneficial properties are attributed to bioactive compounds, including phenolic acids, procyanidins, and abundant anthocyanin pigments, with the greatest impact on blueberry health functionality^20,21^.

Recently, more attention has been paid to the antioxidants contained in these fruits because multiple studies have revealed that their high intake appears to be positively correlated with numerous health benefits^22,23^. They can enhance vision^24^ and provide protection against cancer^25^, diabetes^26^, obesity^27^, and neurodegenerative conditions such as macular degeneration^28^. Additionally, they help prevent osteoporosis, reduce hyperlipidemia and hypertension, and lower the risk of heart disease^29–31^. These effects are attributed to their ability to promote the regeneration of wounds, as well as their antioxidant, anti-inflammatory, and anti-angiogenic properties^32–34^.

In particular, the bioactive compounds of blueberries reduce oxidative damage by decreasing the production of ROS and lipid peroxidation, as well as increasing the activity of antioxidant enzymes such as superoxide dismutase (SOD) and catalase (CAT). Additionally, anthocyanins also act by modulating the NF-κB pathway, thereby reducing the production of inflammatory markers in vitro, such as interleukins, cyclooxygenase-2 (COX2), and inducible nitric oxide synthase (iNOS). These studies on the antioxidant activities of blueberries have mainly emphasized their free radical scavenging ability in vitro^35–37^, while research on their impact at the metabolic level remains limited. Thus, further investigation into the protective effects of blueberries against oxidative damage at the biochemical level is crucial.

Cell metabolomics is emerging as a powerful approach for analyzing metabolic pathways and interactions at the cellular level^38,39^. Current metabolomics techniques enable the profiling of a broad spectrum of metabolites within cells, offering detailed insights into the biochemical processes that drive cellular function and dysfunction.

This approach is increasingly being used in cutting-edge fields such as systems biology and precision medicine to unravel complex metabolic networks and identify biomarkers for disease, and support drug development^40–44^.

In this study, we first conducted an analytical characterization of a blueberry extract (BLUBE) through UHPLC-PDA-ESI-Orbitrap-MS/MS analysis and then evaluated its antioxidant properties by assessing radical scavenger activity with DPPH, FRAP and ABTS assays.

Furthermore, we corroborated the potential antioxidant and cytoprotective properties of BLUBE against H_2_O_2_-induced oxidative damage in IEC-6 intestinal cells, evaluating different cellular parameters, such as ROS, RNS (reactive nitrogen species), clonogenic potential, metabolic activity, wound healing, hypodiploid nuclei and stress fibers organization. Finally, a HR-MS-based metabolomics-based approach was employed to deeply analyze the biochemical response following cell treatment, revealing the protective effect of the extract at the molecular level. Thanks to chemometric approaches, which improved data preprocessing, minimized systematic biases, and enhanced the reliability of metabolomic analysis, we accurately identified differential metabolites and key metabolic pathways, providing deeper insights into the metabolic alterations induced by BLUBE and its protective role against oxidative stress in IEC-6 cells.

## Results

### Total phenolic and flavonoid contents

The extract was initially subjected to the Folin-Ciocalteu assay to determine its phenolic content while the aluminum chloride assay to assess its flavonoid content, using a final concentration of 50 μg/mL.

TPC was quantified based on the regression equation derived from the calibration standard curve of gallic acid (y = 0.0015x − 0.149; R^2^ = 99.96%), and the result was expressed in gallic acid equivalents (GAEs), yielding a value of 86.66 mg GAE/g (Fig. S1).

TFC was determined by extrapolating the regression equation from the rutin calibration standard curve (y = 0.0005x—0.0584; R2 = 99.97%) and expressed in rutin equivalents (REs), with a concentration of 31.06 RE/g (Fig. S1).

### LC–MS/MS screening

Identification and semi-quantification of bioactive compounds in BLUBE were conducted using RP-UHPLC-PDA-ESI–MS/MS. The putative identification of bioactive compounds was carried out by comparing their UV − Vis absorbance, mass fragmentation analysis, and retention times (Table 1). Semi-quantitative analysis revealed that BLUBE is a rich source of phenolic acids, flavonoids, and anthocyanins (Table 2).

**Table 1:** Putatively identified compounds from BLUBE by UHPLC-PDA-ESI-Orbitrap-MS/MS analysis.

Peak	RT (min)	Name	Formula	m/z	MS^2^	Error (ppm)	Adduct	References
Anthocyanins								
22	8.04	Delphinidin hexoside	C_21_ H_20_ O_12_	465.1019	303.05; 304.05; 91.04; 61.03	− 0.3	[M + H]^+1^	[^48^]
30	11.43	Petunidin hexoside	C_22_ H_22_ O_12_	479.1175	317.07	− 1.9	[M + H]^+1^	^48^
31	11.68	Peonidin hexoside	C_22_ H_23_ O_11_	463.123	301.07; 286.05; 196.93	− 1.15	[M + H]^+1^	^49,50^
33	12.19	Cyanidin hexoside	C_21_ H_20_ O_11_	449.1074	317.07; 318.07; 287.06	− 1.06	[M + H]^+1^	^47,48^
35	13.07	Peonidin hexoside	C_22_ H_22_ O_11_	463.1227	301.07; 302.07; 286.05	− 1.78	[M + H]^+1^	^49–51^
36	13.33	Malvidin hexoside	C_23_H_25_O_12_	493.1333	331.08; 332.08; 315.06	− 1.62	[M + H]^+1^	^49^
37	13.56	Dihydromyricetin	C_15_ H_12_ O_8_	319.0459	183.03; 139.04; 153.02	3.31	[M-H]^−1^	^51,52^
39	14.55	Malvidin hexoside (I)	C_23_H_25_O_12_	493.1333	331.08; 332.08; 315.05	− 1.56	[M + H]^+1^	^49^
42	15.32	Malvidin arabinoside	C_22_ H_23_ O_11_	463.1229	331.08; 301.07; 332.09	− 1.27	[M + H]^+1^	^49^
47	16.86	Cyanidin hexoside (I)	C_21_ H_20_ O_11_	449.107	287.13	− 1.22	[M + H]^+1^	^48^
54	19.84	Delphinidin hexoside (I)	C_21_ H_20_ O_12_	465.1018	303.05; 304.05; 91.04; 61.03	− 2.04	[M + H]^+1^	^48,51,53^
56	20.78	Delphinidin hexoside (I)	C_21_ H_20_ O_12_	465.1021	303.05; 412.28; 85.03	− 1.38	[M + H]^+1^	^48,51,53^
72	25.91	Petunidin hexoside (I)	C_22_ H_22_ O_12_	479.1178	317.07; 333.06; 85.03	− 1.3	[M + H]^+1^	^48^
76	26.73	Malvidin arabinoside	C_23_ H_24_ O_13_	507.1143	334.05; 345.06; 273.04	1.86	[M-H]^−1^	^48^
Flavonoids								
11	5.87	Myricetin	C_15_ H_10_ O_8_	319.0444	301.03; 273.04; 235.02	− 1.32	[M + H]^+1^	^48^
13	6.15	Myricetin hexoside	C_21_ H_20_ O_13_	479.083	299.02; 271.02; 191	2.07	[M-H]^−1^	^48,51^
14	6.18	Myricetin arabinofuranoside	C_20_ H_18_ O_12_	451.0868	301.03; 137.02; 73.03	− 0.78	[M + H]^+1^	^51,54^
16	6.38	Myricetin (I)	C_15_ H_10_ O_8_	319.0444	301.03; 273.04; 239.03	− 1.53	[M + H]^+1^	^48^
17	6.46	Myricetin pentoside	C_20_ H_18_ O_12_	449.0726	299.02; 191; 149.02	2.42	[M-H]^−1^	^47^
19	6.65	Myricetin hexoside (I)	C_21_ H_20_ O_13_	479.0829	299.02; 191; 231.03	1.94	[M-H]^−1^	^48^
23	8.3	Kaempferol hexoside	C_21_ H_20_ O_11_	449.1071	287.05; 288.06; 214.6	− 1.64	[M + H]^+1^	^47,51^
25	8.79	Quercetin arabinoside	C_20_ H_18_ O_11_	435.0916	303.05; 304.05; 172.37	− 1.46	[M + H]^+1^	^55^
28	9.56	Kaempferol hexoside (I)	C_21_ H_20_ O_11_	449.1071	287.05; 288.06; 171.49	− 1.64	[M + H]^+1^	^47,51^
29	10.2	Kaempferol arabinofuranose (Juglanin)	C_20_ H_18_ O_10_	419.0966	287.05; 288.06; 160.16	− 1.46	[M + H]^+1^	^51,56^
43	16.05	3',5,6-Trihydroxy-3,4',7,8-tetramethoxyflavone 3-glucoside	C_25_ H_28_ O_14_	551.1403	179.04; 161.02; 135.05	1.54	[M-H]^−1^	^51^
44	16.1	Myricetin hexoside (II)	C_21_ H_20_ O_13_	481.0971	319.04; 91.04; 61.03	− 1.14	[M + H]^+1^	^48^
45	16.5	Myricetin hexoside (III)	C_21_ H_20_ O_13_	479.083	316.02; 317.03; 179	1.94	[M-H]^−1^	^47^
46	16.61	Myricetin hexoside (IV)	C_21_ H_20_ O_13_	481.097	319.04; 85.03; 127.04	− 1.32	[M + H]^+1^	^48^
48	17.01	Myricetin hexoside (V)	C_21_ H_20_ O_13_	479.083	316.02; 317.03; 179	2	[M-H]^−1^	^47^
49	17.31	Medicarpin 3-O- (6'-malonylglucoside)	C_25_ H_26_ O_12_	519.1487	147.04; 175.04; 193.05; 119.05	− 1.91	[M + H]^+1^	^51,57^
51	18.53	Medicarpin 3-O- (6'-malonylglucoside) (I)	C_25_ H_26_ O_12_	519.1489	147.04; 175.04; 193.05; 119.05	− 1.44	[M + H]^+1^	^51,57^
52	19.36	Medicarpin 3-O- (6'-malonylglucoside) (II)	C_25_ H_26_ O_12_	519.1486	147.04; 175.04; 193.05; 119.05	− 1.21	[M + H]^+1^	^51,57^
55	20.23	Quercetin hexoside	C_21_ H_20_ O_12_	463.0879	300.03; 301.04; 179	1.77	[M-H]^−1^	^47,55,56^
57	20.89	Quercetin glucuronide	C_21_ H_18_ O_13_	479.0813	303.05; 304.05; 113.02; 85.03	− 1.54	[M + H]^+1^	^53,58^
58	21.15	Quercetin hexoside (I)	C_21_ H_20_ O_12_	463.088	300.03; 301.04; 179	2.63	[M-H]^−1^	^48,55,56^
59	21.59	Laricitrin hexoside	C_22_ H_22_ O_13_	495.1129	333.06; 91.04; 375.07	− 0.66	[M + H]^+1^	^48,51^
60	21.9	Quercetin glucuronide (I)	C_21_ H_18_ O_13_	477.0675	301.04; 179; 151	2.26	[M-H]^−1^	^57^
61	22.01	Laricitrin hexoside (I)	C_22_ H_22_ O_13_	495.1128	333.06; 85.03; 318.04	− 1.06	[M + H]^+1^	^48,51^
62	22.38	Laricitrin hexoside (II)	C_22_ H_22_ O_13_	493.0989	330.04; 331.05; 315.01	2.52	[M-H]^−1^	^48^
63	22.65	Quercetin arabinoside (I)	C_20_ H_18_ O_11_	435.0916	303.05; 73.03; 369.06	− 1.32	[M + H]^+1^	^55^
65	23.06	Quercetin pentoside	C_20_ H_18_ O_11_	433.0776	300.03; 301.04; 271.03	2.47	[M-H]^−1^	^48,51^
66	23.25	Medicarpin 3-O-(6'-malonylglucoside) (III)	C_25_ H_26_ O_12_	519.1489	147.04; 175.04; 119.05	− 1.61	[M + H]^+1^	^51,57^
69	24.24	Trilobatin	C_21_ H_24_ O_10_	435.1297	273.08; 167.04; 179.04	2.59	[M-H]^−1^	^48,51^
70	24.6	Quercetin	C_15_ H_10_ O_7_	303.0495	275.05; 165.02; 241.27	− 1.47	[M + H]^+1^	^55^
71	24.99	Quercetin rhamnoside (Quercitrin)	C_21_ H_20_ O_11_	447.0931	300.03; 301.04; 151	2.13	[M-H]^−1^	^47^
75	26.43	Syringetin hexoside	C_23_ H_24_ O_13_	509.1283	347.08; 91.04; 291.09	− 1.41	[M + H]^+1^	^47,59^
77	26.8	Syringetin hexoside (I)	C_23_ H_24_ O_13_	509.1281	347.08; 85.03; 287.05	− 1.68	[M + H]^+1^	^47,59^
80	27.79	Patuletin-3-methoxy-4′-glucuronoide	C_23_ H_22_ O_14_	523.1075	347.08; 85.03; 113.02	− 1.37	[M + H]^+1^	^51,60^
84	30.02	Liquiritin 2’'-apioside	C_26_ H_30_ O_13_	533.1644	161.06; 175.04; 179.07; 147.04	− 1.79	[M + H-H_2_O]^+1^	^51^
85	30.21	Liquiritin 2’'-apioside (I)	C_26_ H_30_ O_13_	549.1612	147.05; 177.06; 191.04	1.8	[M-H]^−1^	^51^
86	31.04	Prunetin 4’-O-glucoside	C_22_ H_22_ O_10_	445.1138	179.04; 135.05; 121.03	2.04	[M-H]^−1^	^51^
Phenolic acids								
3	2.41	Dihydroxybenzoic acid	C_7_ H_6_ O_4_	153.0193	109.03; 108.02; 81.03	− 1.77	[M-H]^−1^	^51,56^
6	4.03	1-O-protocatechuyl-β-xylose	C_12_ H_14_ O_8_	285.0617	152.01; 108.02; 153.02	4.15	[M-H]^−1^	^45^
8	4.42	Dihydroferulic acid glucuronide	C_16_ H_20_ O_10_	371.0985	163.04; 119.05; 164.04	3.25	[M-H]^−1^	^51,56^
9	4.66	Caffeoylhexose	C_15_ H_18_ O_9_	341.0878	179.04; 135.05; 84.17	3.38	[M-H]^−1^	^45,51^
10	5.77	Caffeic acid hexoside dimer	C_30_ H_36_ O_18_	685.1959	163.04; 181.05	− 1.95	[2 M + H]^+^	^45,51^
12	5.91	Caffeoylhexose (I)	C_15_ H_18_ O_9_	341.0876	179.04; 135.05; 54.8	2.75	[M-H]^−1^	^45,51^
15	6.31	Dihydroxybenzoic acid (I)	C_7_ H_6_ O_4_	155.0337	127.04; 113.96; 69	− 1.31	[M + H]^+1^	^61^
18	6.5	Ferulic acid	C_10_ H_10_ O_4_	177.0543	145.03; 117.03; 149.06	− 1.47	[M + H-H_2_O]^+1^	^62^
20	6.88	Chlorogenic acid	C_16_ H_18_ O_9_	355.1017	163.04; 145.03; 337.09	− 1.85	[M + H]^+1^	^48^
21	7.01	Chlorogenic acid dimer	C_16_ H_18_ O_9_	707.1825	191.06; 353.09; 161.02	2.73	[2 M-H]^−1^	^51,63^
27	9.33	Catechin	C_15_ H_14_ O_6_	289.0717	245.08; 203.07; 125.02	3.53	[M-H]^−1^	^56^
32	11.77	Chlorogenic acid (I)	C_16_ H_18_ O_9_	335.0774	179.03; 135.05; 161.02	3.64	[M-H]^−1^	^51,55^
40	14.73	Ferulic acid hexoside	C_16_ H_20_ O_9_	355.1033	193.05; 161.03; 100.38	2.48	[M-H]^−1^	^51,56^
41	15.28	3-Feruloylquinic acid	C_17_ H_20_ O_9_	367.1034	179.04; 135.05; 161.02	2.82	[M-H]^−1^	^62^
64	22.88	Coumarinic acid hexoside	C_15_ H_18_ O_8_	309.0965	147.04; 127.04; 165.05	− 1.34	[M + H-H_2_O]^+1^	^51^
73	26.23	Coumarinic acid hexoside (I)	C_15_ H_18_ O_8_	309.0961	147.04; 165.05; 127.04	− 1.84	[M + H-H_2_O]^+1^	^51^
78	27.12	Coumaric acid	C_9_ H_8_ O_3_	147.0438	119.05; 91.05; 148.05	− 1.26	[M + H-H_2_O]^+1^	^61^
81	28.07	Coumarin	C_9_ H_6_ O_2_	147.0438	119.05; 91.05; 148.05	− 1.66	[M + H]^+1^	^61^
83	28.73	Chicoric acid	C_21_ H_30_ O_12_	473.1665	249.06; 179.11; 267.07; 149.05	2.24	[M-H]^−1^	^45^
Terpene glycosides								
2	1.75	Monotropein	C_16_ H_22_ O_11_	389.1086	165.06; 183.07; 209.05	2.15	[M-H]^−1^	^51,64^
38	14.27	Deoxyloganic acid	C_16_ H_24_ O_9_	359.1346	153.09; 197.08; 89.02; 59.01	2.83	[M-H]^−1^	^51^
50	17.58	Vaccinoside	C_25_ H_28_ O_13_	535.1452	163.04; 147.05; 191.03	1.09	[M-H]^−1^	^48,51^
53	19.62	Vaccinoside (I)	C_25_ H_28_ O_13_	535.1454	163.04; 147.05; 191.04	1.44	[M-H]^−1^	^48,51^
67	23.58	Vaccinoside (II)	C_25_ H_28_ O_13_	535.1455	163.04; 147.05; 191.04	1.55	[M-H]^−1^	^48,51^
68	23.97	Vaccinoside (III)	C_25_ H_28_ O_13_	535.1456	163.04; 165.06; 145.03; 89.02	1.89	[M-H]^−1^	^48,51^
Norisoprenoids								
24	8.6	Vomifoliol hexoside	C_19_ H_30_ O_8_	431.1922	205.12; 385.19	2.31	[M-H]^−1^	^65^
26	8.85	Vomifoliol hexoside (I)	C_19_ H_30_ O_8_	431.1923	205.12; 385.19	2.52	[M-H]^−1^	^65,66^
34	12.77	Vomifoliol hexoside (II)	C_19_ H_30_ O_8_	431.1923	205.12	2.38	[M-H]^−1^	^65,67^
Organic compounds								
1	1.46	Styrene	C_8_H_8_	105.0697	79.05	− 1.81	[M + H]^+1^	^51,68^
5	3.37	6''-O-Acetylholocalin	C_16_H_19_NO_8_	354.1178	174.05; 146.06; 192.07	− 1.19	[M + H]^+1^	^51,69^
7	4.33	6''-O-Acetylholocalin (I)	C_16_H_19_NO_8_	354.1178	174.05; 146.06; 188.07	− 1.54	[M + H]^+1^	^51,69^
4	3.26	Arbutin	C_12_ H_16_ O_7_	271.0824	109.03; 110.03; 149.04	4.2	[M-H]^−1^	^62^
74	26.36	Phenethyl rutinoside	C_20_ H_28_ O_9_	411.1659	145.03; 163.04; 119.05	2.35	[M-H-H_2_O]^−1^	^50,51^
79	27.22	Phenethyl rutinoside (I)	C_20_ H_28_ O_9_	411.1659	145.03; 163.04; 119.05	2.2	[M-H-H_2_O]^−1^	^50,51^
82	28.23	Phenethyl rutinoside (II)	C_20_ H_28_ O_9_	411.1658	145.03; 163.04; 119.05	1.98	[M-H-H_2_O]^−1^	^50,51^

**Table 2:** Semi-quantitative analysis of the major compounds identified in BLUBE.

Peak	Anthocyanins	mg CynGluE g^−1^ dw
22	Delphinidin hexoside	0.01 ± 0.001
31	Peonidin hexoside	3.74 ± 0.56
33	Cyanidin hexoside	0.18 ± 0.01
35	Peonidin hexoside	0.106 ± 0.004
42	Malvidin arabinoside	0.016 ± 0.001
47	Cyanidin hexoside	0.033 ± 0.002
56	Delphinidin hexoside	0.885 ± 0.003
54	Delphinidin hexoside	4.22 ± 0.01
72	Petunidin hexoside	0.277 ± 0.003
30	Petunidin hexoside	< LOQ
36	Malvidin hexoside	< LOQ
39	Malvidin hexoside	< LOQ

	Phenolic acids	mg CGAE g^−1^ dw
3	Dihydroxybenzoic acid	4.16 ± 0.03
6	1-O-protocatechuyl-β-xylose	1.09 ± 0.02
9	Caffeoylhexose	1.41 ± 0.01
12	Caffeoylhexose	18.10 ± 0.72
21	Chlorogenic acid dimer	57.91 ± 1.28
27	Catechin	2.33 ± 0.02
32	Chlorogenic acid	0.117 ± 0.002
40	Ferulic acid hexoside	2.48 ± 0.05
41	3-Feruloylquinic acid	1.59 ± 0.02
83	Chicoric acid	1.18 ± 0.01
8	Dihydroferulic acid glucuronide	< LOQ

	Flavonoids	mg QE g^−1^ dw
13	Myricetin hexoside	0.59 ± 0.03
17	Myricetin pentoside	0.32 ± 0.01
19	Myricetin hexoside	0.58 ± 0.01
43	3′,5,6-Trihydroxy-3,4′,7,8-tetramethoxyflavone 3-glucoside	0.48 ± 0.01
45	Myricetin hexoside	0.79 ± 0.03
48	Myricetin hexoside	0.73 ± 0.01
55	Quercetin hexoside	4.49 ± 0.32
58	Quercetin hexoside	0.98 ± 0.01
62	Laricitrin hexoside	0.33 ± 0.01
65	Quercetin pentoside	0.590 ± 0.003
71	Quercetin rhamnoside (Quercitrin)	1.27 ± 0.01
85	Liquiritin 2′′-apioside	1.16 ± 0.03
86	Prunetin 4′-O-glucoside	0.84 ± 0.01
60	Quercetin hexoside	< LOQ
69	Trilobatin	< LOQ

Data expressed as mean (mg g^−1^ dried extract) ± deviation standard (n = 3)

CynGluE: Cyanidin 3-glucoside Equivalents; CGAE: Chlorogenic acid Equivalents; QE: Quercetin Equivalents

Among the investigated analytes, special attention was given to peak **10**, which was putatively identified as a dimer of caffeic acid hexoside. Compound **10** exhibited a [M + H]^+^ ion at *m/z* 685, consistent with a molecular formula of C_30_H_36_O_18_. MS^2^ fragmentation produced an ion at *m/z* 343 indicating a dimeric structure. Further fragmentation yielded an ion at *m/z* 181, corresponding to the protonated caffeic acid, and an ion at *m/z* 163, attributed to the hexoside moiety. To the best of our knowledge, while hydroxycinnamic acid dimers have been described in various studies, this particular compound has not yet been reported in *Vaccinium myrtillus L.*^45,46^.

Compound **21** was putatively identified as a chlorogenic acid dimer and was quantified as the most abundant compound in the extract, with a concentration of 57.91 ± 1.28 mg g⁻^1^ dw. The precursor ion [M–H]⁻ at *m/z* 707 generated product ions at *m/z* 353, indicating the loss of a chlorogenic acid monomer, and at *m/z* 191, corresponding to the subsequent loss of a hexoside moiety.

Peaks **9** and **12** were identified as two of the most abundant compounds in the extract, with concentrations of 1.41 ± 0.01 mg g⁻^1^ dw and 18.10 ± 0.72 mg g⁻^1^ dw, respectively. Both were putatively identified as caffeoyl hexose. These compounds exhibited a deprotonated [M–H]⁻ ion at *m/z* 341, with MS^2^ fragmentation producing a key ion at *m/z* 179. This fragment resulted from the loss of a hexoside moiety [M–H–162]⁻, corresponding to a caffeic acid residue^45^.

Among the flavonoids, the most abundant compounds were peak **55**, with a concentration of 4.49 ± 0.32 mg g⁻^1^ dw, and peak **71**, with a concentration of 1.27 ± 0.01 mg g⁻^1^ dw. These compounds were putatively identified as quercetin hexoside and quercetin rhamnoside (quercitrin), respectively. Both compounds exhibited characteristic fragmentation patterns indicative of the loss of a sugar group, yielding quercetin aglycone. Quercetin hexoside showed a precursor ion at *m/z* 463 with an MS^2^ fragment at *m/z* 301 [M–H–162]⁻, corresponding to the loss of a hexoside moiety. Similarly, quercitrin showed a [M–H]⁻ ion at *m/z* 447 and a product ion at *m/z* 301 [M–H–146]⁻, corresponding to the loss of a rhamnoside moiety^47^.

Among anthocyanin, the major compound identified was delphinidin hexoside (peak **54**), with a concentration of 4.22 ± 0.01 mg g^−1^ dw. Additional isomers, putatively identified as compounds **22** and **56**, were characterized by a precursor ion at *m/z* 465. The MS^2^ fragmentation pattern of these isomers revealed the loss of a hexoside group, resulting in a characteristic fragment at *m/z* 303, corresponding to the delphinidin aglycone^48^.

Compounds **31** and **35** were identified as isomers of peonidin hexoside, with an [M + H]^+^ ion at *m/z* 463. Peonidin aglycone was observed as an MS^2^ fragment ion at *m/z* 301, resulting from the loss of 162 Da, corresponding to the hexoside moiety^48^. These compounds were quantified at 3.74 ± 0.56 mg g⁻^1^ dw and 0.106 ± 0.004 mg g⁻^1^ dw, respectively.

### Chelating properties and in vitro antioxidant activity

The antioxidant activity assay of BLUBE was performed with DPPH and ABTS tests. The data were expressed as a Trolox equivalent antioxidant capacity (TEAC, mg TXE/g) using a Trolox calibration curve in the range 0.025–0.2 mg. The extract was tested in a concentration range of 25–200 μg/mL, and radical scavenging activity was calculated using the formulas reported in the experimental section. The results showed a dose-dependent radical-scavenging potential for DPPH (TEAC = 469.53 ± 3.01 mg TXE/g) and ABTS assays (TEAC = 409.68 ± 2.69 mg TXE/g), suggesting good antioxidant activity for the extract compared to trolox, used as positive control.

Furthermore, the FRAP method was performed to corroborate these data. FRAP method is based on the ability of an antioxidant to reduce Fe^3+^ to Fe^2+^ ions in the presence of TPTZ, forming an intense blue Fe^2+^-TPTZ complex. BLUBE was tested in a concentration range of 25–200 μg/mL, and radical scavenging activity was calculated and quantified as 2.95 mg of trolox equivalents per gram of dry weight (mg TXE g^−1^ dw) (Fig. S1).

Metal binding studies were performed to further validate these findings. The results show that BLUBE exerts its antioxidant activity through the chelation of iron, zinc and copper ions supporting the high polyphenolic content and the antioxidant effects observed in previous assays (Fig. S1).

### In-cell cytoprotection

**Fig. 1:**
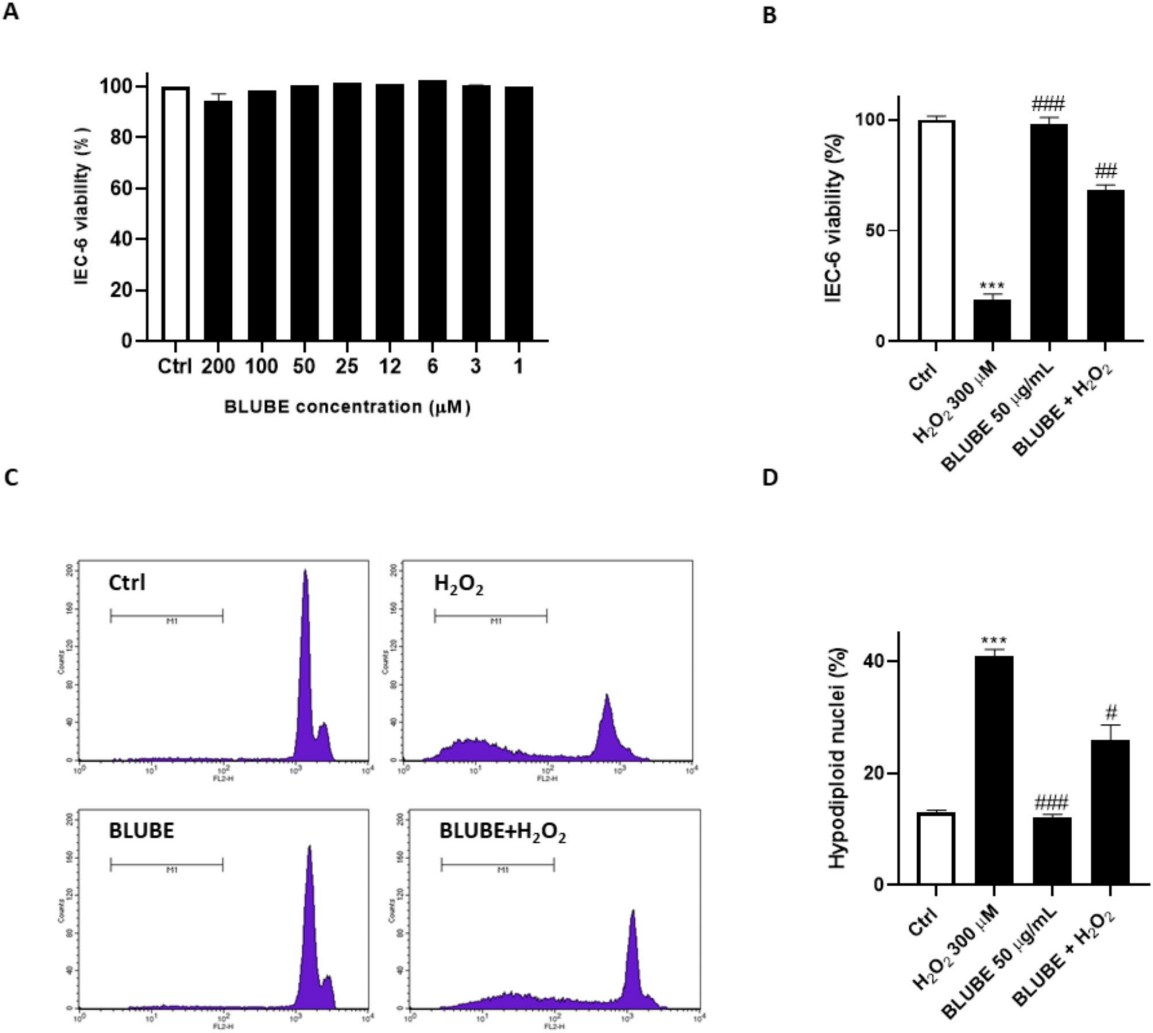
In-cell cytoprotection exerted by BLUBE. (**A**) IEC-6 cells were treated with BLUBE in the range of 1–200 µg/mL. (**B**) Protective effect of the BLUBE against cytotoxicity on H_2_O_2_-induced mitochondrial activity using MTT reagent. Changes in viability were calculated as percentage of viable cells in treated cultures versus untreated ones (set as 100% of viability). (**C**, **D**) Protective effect of the BLUBE against cytotoxicity on H_2_O_2_-induced nuclear fragmentation by PI staining. Results are showed as mean ± standard deviation (SD) from three independent experiments. *** denote *p* < 0.001 vs. Ctrl; #, ## and ### denote respectively *p* < 0.05, *p* < 0.01 and *p* < 0.001 vs. H_2_O_2._

The antioxidant activity of BLUBE was also evaluated in cells. Firstly, the extract was tested for its effect on IEC-6 cell viability. Our experimental findings demonstrated that varying concentrations (6–200 μg/mL) of the extract did not cause a decrease in cell viability in the examined cell lines after 24 h of treatment (Fig. 1A).

Based on these results, a concentration of 50 μg/mL was selected as the optimal concentration for subsequent intracellular antioxidant assessments. To test the protective effect of the extract in H_2_O_2_-stimulated IEC-6 cells, we performed an MTT assay, incubating cells with the extract simultaneously with H_2_O_2_ (300 µM). After 24 h, H_2_O_2_ induced a significant reduction in metabolic activity (18.76 ± 2.51% of cell viability, *p* < 0.001 vs. Ctrl). However, when the extract was present alongside H_2_O_2_, it significantly reduced cell mortality compared to cells treated with H_2_O_2_ alone, preserving cell viability at 68.30 ± 2.25% (*p* < 0.01 vs. H_2_O_2_) (Fig. 1B).

We further assessed the protective activity of BLUBE against H_2_O_2_-induced oxidative damage by flow cytometry using PI staining. Treatment with hydrogen peroxide for 24 h increased the percentage of hypodiploid nuclei from 13.12 ± 0.40% (Ctrl) to 41.65 ± 1.14% (*p* < 0.001 vs. Ctrl). Pre-treatment with 50 µg/mL of BLUBE reduced the number of cells undergoing nuclear fragmentation to 26.34 ± 2.65% (*p* < 0.05 vs. H_2_O_2_) (Fig. 1C,D) confirming the beneficial effects of the extract on the survival of IEC-6 cells.

**Fig. 2:**
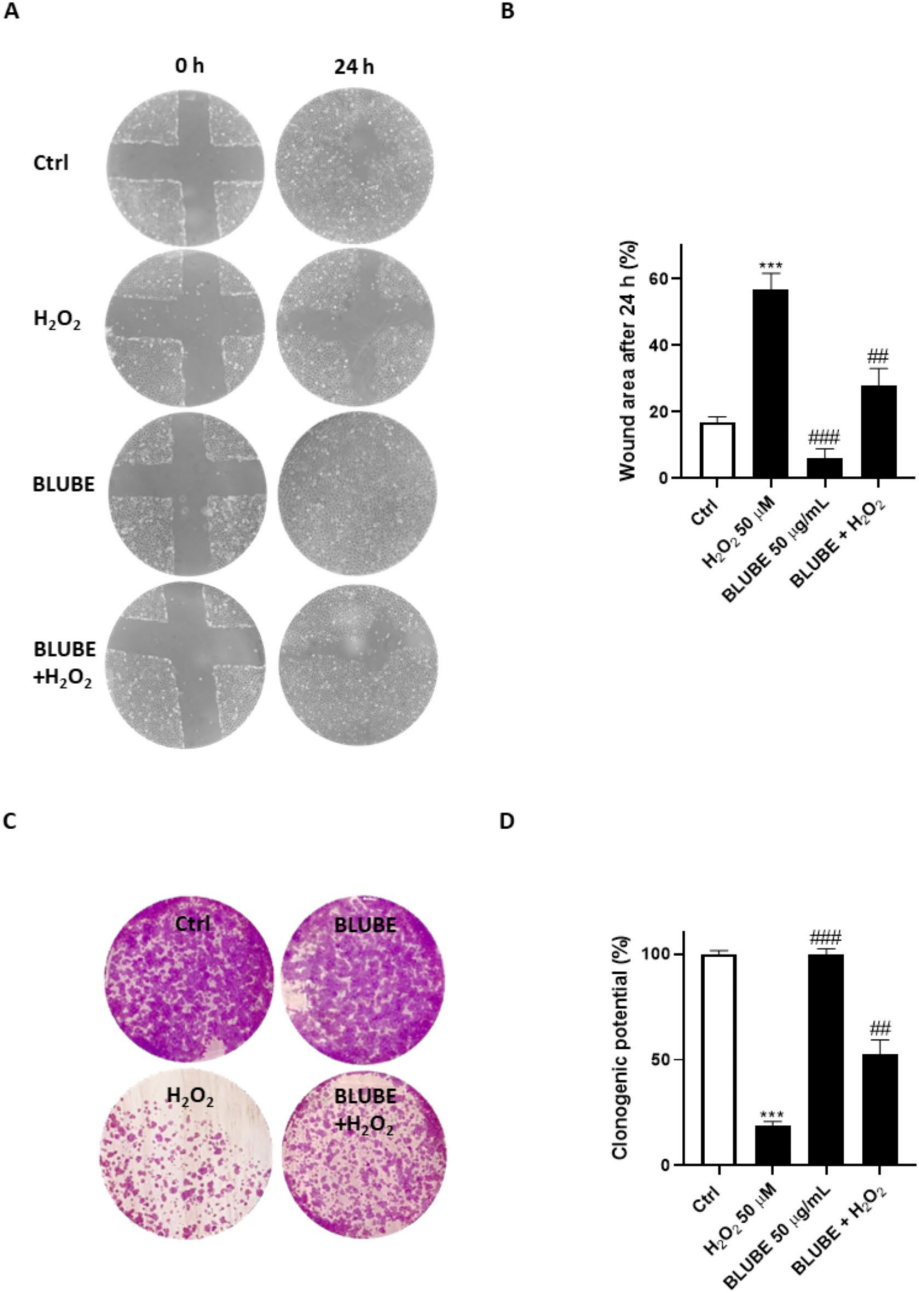
Protective effects of BLUBE on long term proliferation and wound healing. (**A**,** B**) Effect of BLUBE (50 µg/mL) on wound repair after a mechanical scratch in IEC-6 cells treated with H_2_O_2_ (50 µM). (**C**, **D**) Effect of the BLUBE (50 µg/mL) against cytotoxicity on H_2_O_2_-induced clonogenic potential. Results are showed as mean ± standard deviation (SD) from three independent experiments. *** denote *p* < 0.001 vs. Ctrl; ## and ### denote respectively *p* < 0.01 and *p* < 0.001 vs. H_2_O_2._

Following oxidative stress-induced intestinal damage, IEC-6 cells undergo division and proliferation to repair the damaged area, which is essential for wound closure. To assess the potential of BLUBE in promoting intestinal regeneration, we investigated its effects on scratch wound healing and cell migration speed. Our results demonstrated that BLUBE (50 µg/mL) significantly enhanced cell migration compared to cells treated with H_2_O_2_ (1 µM) (*p* < 0.001 vs. H_2_O_2_; Fig. 2A,B). In addition, the clonogenic potential of IEC-6 cells was evaluated to further understand the impact of BLUBE on long-term proliferation. A low subtoxic dose of H_2_O_2_ (50 μM) significantly reduced colony formation after 10 days of incubation. However, treatment with BLUBE (6 µg/mL) reversed these effects, leading to an increase in long-term cell proliferation, with a growth rate of 53.46 ± 9.23% (Fig. 2C,D).

### In-cell antioxidant activity

**Fig. 3:**
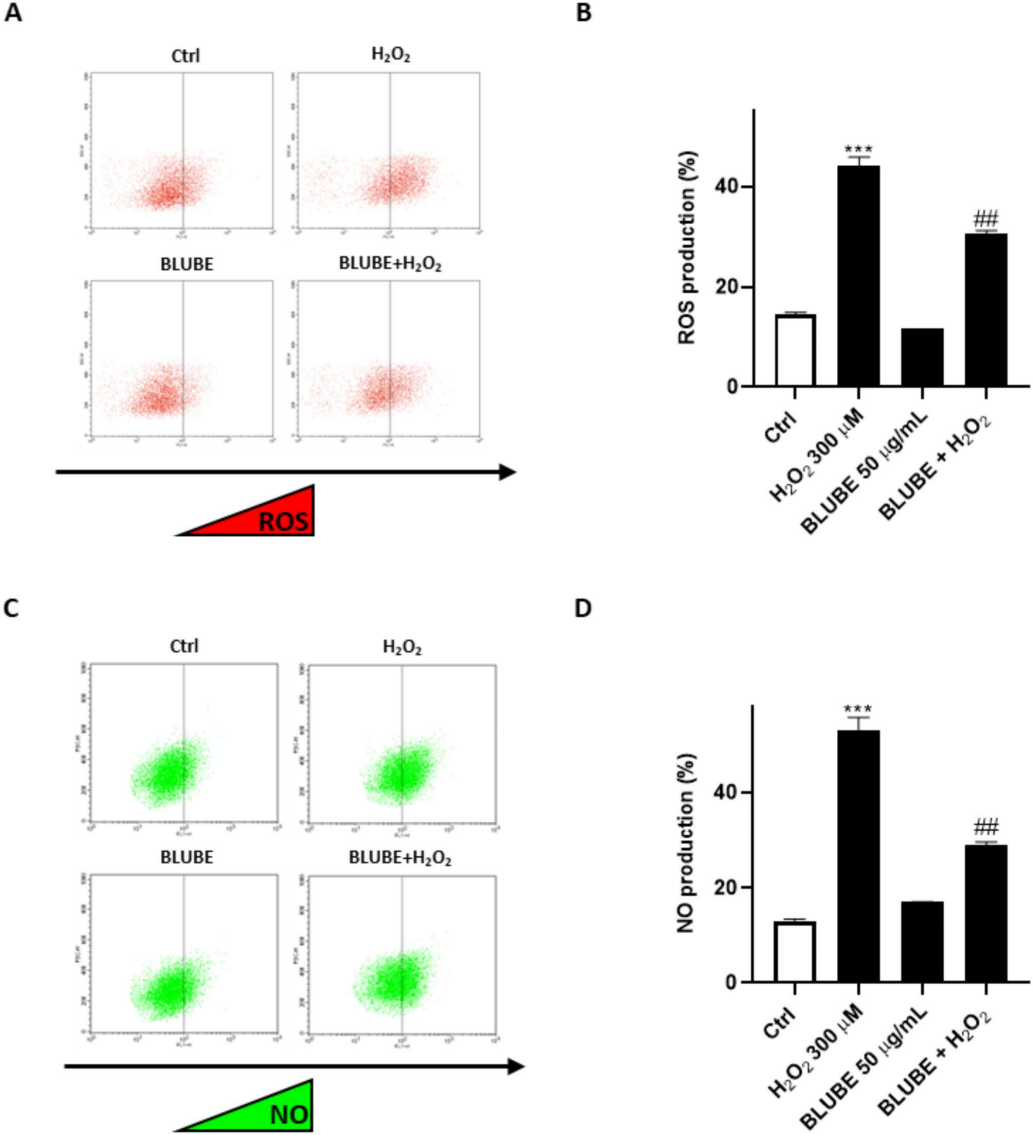
Antioxidant effects of BLUBE on IEC-6 cells. (**A**, **B**) ROS formation was assessed through DCFH-DA probe, while (**C**, **D**) RNS formation was assessed through DAF-FM-DA probe, both by cytofluorimetric technique. Results are shown as mean ± standard deviation (SD) from three independent experiments. *** denote *p* < 0.001 vs. Ctrl; ## denote *p* < 0.01 vs. H_2_O_2._

Next, we aimed to investigate the mechanisms underlying the protective effect exerted by the extract. To this end, we firstly evaluated H_2_O_2_-induced oxidative stress by assessing intracellular ROS levels using the DCFH-DA assay through flow cytometry. Our results showed that H_2_O_2_ significantly increased ROS production in IEC-6 cells (44.08 ± 1.79%; *p* < 0.001 *vs*. Ctrl). However, when BLUBE was added to cells, ROS release was significantly inhibited (30.55 ± 0.67%; *p* < 0.01 vs. H_2_O_2_), confirming the antioxidant properties of blueberry in neutralizing ROS after 24 h of treatment (Fig. 3A,B).

Furthermore, we detected H_2_O_2_-induced nitrosative stress by measuring intracellular nitric oxide (NO) levels using the DAF-FM assay through flow cytometry. Although NO is a weak oxidant, it can react with superoxide to form peroxynitrite. Both NO and peroxynitrites can generate nitrite and nitrate ions, which accumulate in cells along with their intermediates, leading to the nitration and nitrosation of biomolecules, thereby disrupting their function. Our results showed that H_2_O_2_ significantly increased NO production in IEC-6 cells (53.77 ± 2.79%; *p* < 0.001 vs. Ctrl). In contrast, BLUBE significantly mitigates the nitrosative stress caused by hydrogen peroxide (29.15 ± 0.65% of NO; *p* < 0.01 vs. H_2_O_2_) (Fig. 3C,D).

### BLUBE protects IEC-6 cells against actin cytoskeleton rearrangement

**Fig. 4:**
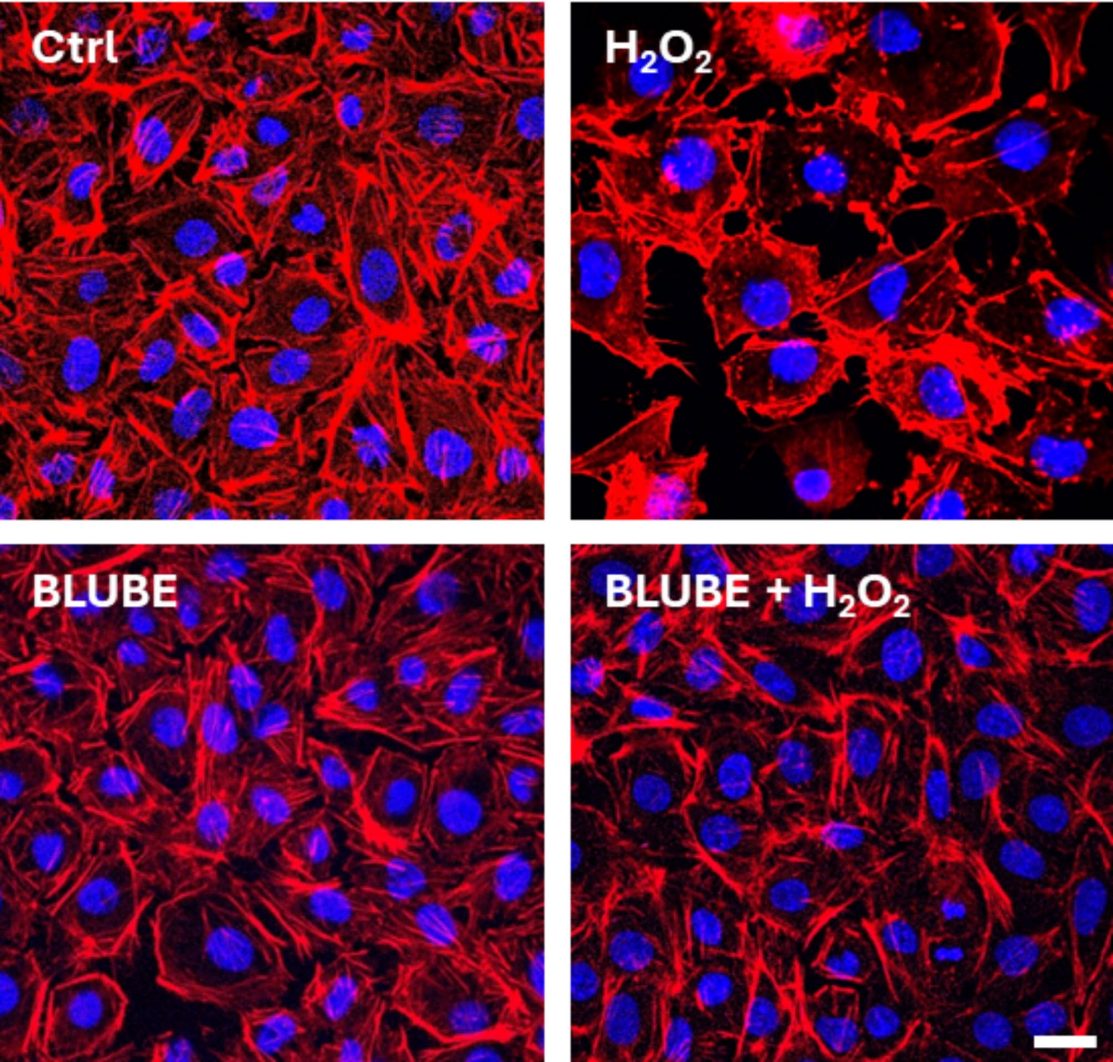
BLUBE prevents actin stress fibers rearrangement in IEC-6 cells. The analysis was performed using confocal microscopy. F-actin was stained with phalloidin (red), and nuclei were stained with Hoechst 33,342 (blue). Scale bar, 10 µm. N = 10.

To explore some of the mechanisms behind the antioxidant protection exerted by BLUBE, we investigated the effect of the extract (50 µg/mL) on actin stress fiber assembly in IEC-6 cells. In control cells, the cytoskeleton and actin fibers were intact and well-organized throughout the cytosol. However, treatment with H_2_O_2_ caused significant actin filament remodeling, disrupting central stress fibers, impairing cell adhesion, and leading to increased cell detachment. In contrast, BLUBE treatment did not affect cell morphology compared to control. When administered together with H_2_O_2_, BLUBE reversed the H_2_O_2_-induced changes in cell phenotype, restoring normal actin structure (Fig. 4).

### Metabolomic analysis

#### Principal component analysis (PCA)

Once we had collected data on the protective activity of our extract, we proceeded with biochemical investigations, using the cell metabolomics approach, to associate the protective phenotypic effects found with metabolic changes.

To investigate experimental variability and identify potential sources of systematic noise, an initial PCA was performed using both technical replicates and QC samples. Prior to PCA, the data were preprocessed to ensure comparability across samples. Specifically, the data were normalized using the median by row method, followed by PQN, with the median spectrum of the control group used as the reference spectrum. This preprocessing step was crucial for minimizing variations unrelated to biological differences, such as differences in sample concentration or instrumental drift.

The PCA analysis revealed that PC2, which explained 13.48% of the total variance, was dominated by experimental artifacts such as batch effects and other systematic biases (Fig. S2). These findings indicated that PC2 captured primarily unwanted variability, suggesting the need to exclude it in order to focus on biologically relevant signals. To address this, PC2 was removed, and the data were reconstructed, as detailed in the “Exploratory Tools: Principal Component Analysis (PCA) and Data Reconstruction” section of the Materials and Methods. This approach aimed to eliminate the noise stemming from technical variability while preserving the underlying biological patterns. The effectiveness of this correction is illustrated in Fig. S3, which shows the scores plot for the non-averaged replicates. The removal of PC2 significantly improved the data structure, mitigating the confounding effects of batch-related variability and other systematic biases. Subsequent to the data reconstruction, technical replicates were averaged to provide a more stable dataset for further analysis.

**Fig. 5:**
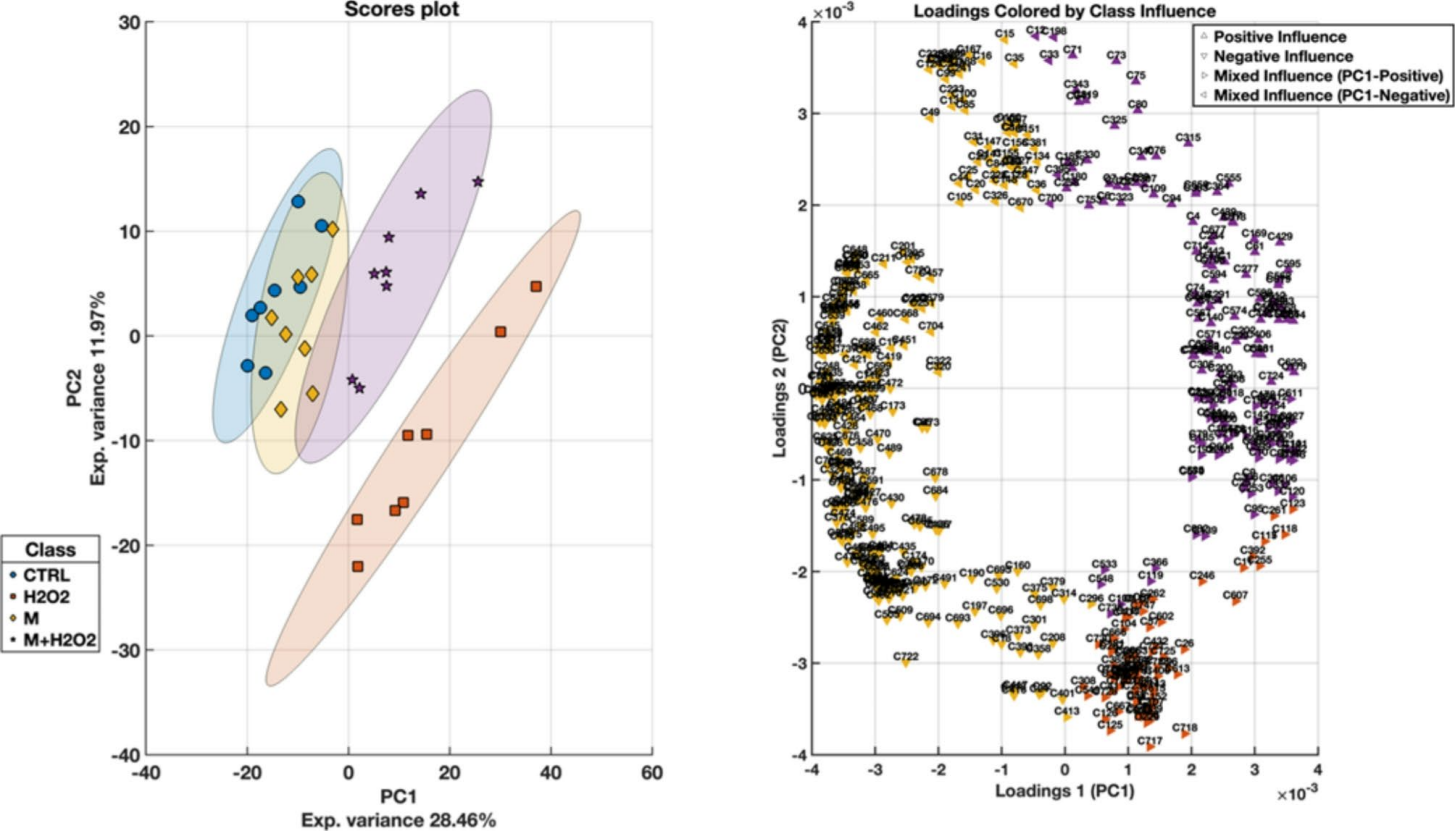
PCA scores and loadings plot after removing PC2, reconstructing the data and averaging technical replicates. (A) PCA scores plot showing the distribution of samples along the first two principal components (PC1 and PC2), which capture 28.46% and 11.97% of the total variance, respectively. The different clusters correspond to Control (CTRL), H_2_O_2_-induced oxidative stress in IEC-6 cells (H_2_O_2_), BLUBE in IEC-6 cells (M), and H_2_O_2_-induced oxidative stress + BLUBE-IEC-6 cells (M + H_2_O_2_). Confidence ellipses (based on Hotelling’s T^2^ statistic for a 95% confidence interval) are overlaid to illustrate the variability within each group. These ellipses represent the spread of the data along the principal axes, showing the direction of maximum variance. (B) PCA loadings plot displaying significant variable influences, colored by class association. Each loading is represented by triangles indicating the direction of influence on PC1 and PC2: upward-facing triangles (▲) denote positive contributions, while downward-facing triangles (▼) denote negative contributions. Mixed influences with PC1-positive are displayed by right-facing triangles (►), and mixed influences with PC1-negative are displayed by left-facing triangles (◄). The colors correspond to different classes, highlighting the relationship between each variable and its respective class. This visualization aids in understanding the key variables driving class separation within the dataset.

In the final PCA model, applied to the reconstructed and averaged dataset, clear stratifications were evident in the data. The scores plot (Fig. 5) revealed a notable tendency for separation between the experimental groups, providing a clear visual representation of their interrelationships. For clarity, different cell treatments were color-coded for clarity: IEC-6 control cells are represented by blue circles, BLUBE-IEC-6 cells by yellow diamonds, H_2_O_2_-induced oxidative stress in IEC-6 cells by orange squares, and H_2_O_2_-induced oxidative stress in BLUBE-IEC-6 cells by purple stars.

To evaluate preliminary differentiation among sample groups, Hotelling’s T^2^ confidence ellipses were added to the score plots. These ellipses visually represent the confidence intervals for each class, calculated independently at a 95.00% confidence level, thereby facilitating the assessment of class separation and the effectiveness of PCA in distinguishing the sample groups. The separation among these groups was most pronounced in the space defined by PC1 and PC2. PC1 accounted for 28.46% of the total variance and played a crucial role in differentiating the experimental groups, while PC2 contributed an additional 11.97% of the variance, further refining this separation. Together, these two principal components explained a substantial portion of the dataset’s variance, offering a valuable framework for understanding the underlying differences in cellular responses to various treatments.

The distinct clustering of IEC-6 cells under H_2_O_2_-induced oxidative stress conditions suggests that the oxidative stress induced by H_2_O_2_ triggered a unique and measurable cellular response. This response was sufficiently different from both control cells and those treated with the BLUBE extract, allowing for its clear distinction in the PCA space. The clear separation implies that oxidative stress likely affects the cellular pathways and mechanisms in a way that is not observed in the untreated or extract-treated cells, making this condition easily identifiable in the scores plot.

Interestingly, when assessing the control samples, including both untreated IEC-6 cells and BLUBE-IEC-6 cells, both were located on the negative side of the PC1 axis. This indicates that, despite some experimental variations, these two groups share significant similarities in their overall cellular profiles. On the same axis, H_2_O_2_-induced oxidative stress in BLUBE-IEC-6 cells exhibited behavior that more closely resembled that of H_2_O_2_-induced oxidative stress in IEC-6 cells, suggesting that the introduction of the extract did not completely mitigate the effects of oxidative stress, at least in terms of its impact on the variables captured by PC1.

However, when considering PC2, a different trend emerged. While PC1 emphasized the similarities between the two H_2_O_2_-induced oxidative stress conditions, PC2 captured nuances in the response of H_2_O_2_-induced oxidative stress in BLUBE-IEC-6 cells, aligning it more closely with the control cells and treated with BLUBE alone (IEC-6 cells and BLUBE-IEC-6 cells). This suggests that, although oxidative stress was a dominant factor, the presence of the BLUBE extract may have influenced certain cellular pathways, leading to a response that, in certain dimensions, more closely resembled that of the controls. This trend highlights the complexity of cellular responses to oxidative stress and potential protective treatments, with the effects of the extract being more subtle or confined to specific components of the cellular response.

The analysis demonstrated that IEC-6 cells exposed to H_2_O_2_-induced oxidative stress formed a distinctly separated cluster from the other experimental groups, highlighting a significant divergence in cellular responses. In contrast, the other sample types, including both control and treated cells, exhibited partial overlap within the subspace defined by the first two principal components. This similarity is likely attributable to the BLUBE extract’s ability to mitigate the effects of H_2_O_2_ or to restore the cellular damage caused by oxidative stress. The extract may exert protective or reparative effects on the cells, leading to a cellular response that is more aligned with that of the untreated controls, despite being exposed to oxidative stress. This observation suggests that BLUBE potentially counteracts the damage induced by H_2_O_2_, helping to repristinate normal cellular function and thereby reducing the distinct separation that would otherwise be expected between stressed and unstressed cells. This aligns with the trend observed in the PCA plot, where cells treated with the extract under oxidative stress show behavior more similar to the controls, particularly in dimensions defined by PC2.

The analysis of loadings in PCA represents the contribution of each original variable to the respective principal components. Figure 5 reports the most relevant loadings selected based on a predefined loading threshold of 0.5 relative to the maximum loadings. This threshold ensures that only those variables that have a substantial contribution to the PCA are visualized, enhancing the interpretability of the results.

The loadings plot illustrates how different variables influence the separation between the experimental conditions. The arrows indicate the type of influence, while the colors point to the group most affected by these variables. Together, this visualization helps identify which variables are driving the differences between groups in the PCA, revealing patterns and insights based on the data structure. In fact, these loadings plot not only shows the direction and type of influence that each variable has on the PCA but also highlights which class each variable most affects. The color coding aligns with the scores plot, reflecting which class is most impacted by that variable. The arrows attached to each variable indicate the type of influence each variable has on the PCA components. Upward-facing triangles (▲) denote positive contributions to both PC1 and PC2, meaning these variables are heavily involved in separating samples in the upper-right quadrant of the scores plot. While, downward-facing triangles (▼) indicate negative contributions along both PC1 and PC2, placing more weight on separating data points in the lower-left quadrant. The mixed influences can be particularly insightful. For instance, they may highlight complex relationships between experimental conditions, revealing that some variables play multifaceted roles in distinguishing between groups. For example, a variable might enhance separation in one principal component while suppressing it in another, reflecting a nuanced biological or chemical interaction.

The mixed influences with PC1-positive and PC2-negative are represented by right-facing triangles (►), likely influencing data points positioned on the positive PC1 axis but lower on the PC2 axis. Specific variables exhibiting this influence include C123, C749 and C718 (Table 3). Conversely, mixed influence variables contributing negatively to PC1 but positively to PC2 are indicated by left-facing triangles (◄), affecting samples that are lower on the PC1 axis and higher on the PC2. Examples of such variables include C688, C633, C240 and C681 (Table 3).

**Table 3:** Loadings and VIP score values of metabolites associated with significant pathways identified in the metabolomic analysis of the different clusters correspond to Control (CTRL), H_2_O_2_-induced oxidative stress in IEC-6 cells (H_2_O_2_), BLUBE in IEC-6 cells (M), and H_2_O_2_-induced oxidative stress-BLUBE-IEC-6 cells (M + H_2_O_2_).

Metabolite ID	Loadings PC1	Loadings PC2	VIP Score	Quadrant	Metabolite	HMDB	RT (min)	Calc. MW	m/z	Chemical Formula	Error (ppm)	Pathway
C123	0.003597256	− 0.001319728	1.185327	►	N-Acylsphingosine	HMDB0011773	0.748	509.47983	510.48709	C_32_H_63_NO_3_	− 1.9	1
C403	−	−	1.366234	−	Sphingosine 1-phosphate	HMDB0000277	3.129	379.24834	380.25561	C_18_H_38_NO_5_P	− 1.11	1
C688	− 0.003185744	0.000440457	1.220676	◄	Hypotaurine	HMDB0000965	4.137	109.01943	110.0267	C_2_H_7_NO_2_S	− 2.96	2
C749	0.00354993	− 0.000771474	1.019291	►	L-Cystine	HMDB0000192	5.24	240.02355	241.03082	C_6_H_12_N_2_O_4_S_2_	− 1.26	4
C671	− 0.00387789	− 0.00037018	1.001649	▼	Glutamic acid (Glutamate)	HMDB0000148	3.996	147.05295	148.06022	C_5_H_9_NO_4_	− 1.42	3
C733	− 0.00379413	− 0.000386708	1.051176	▼	Ethanolamine phosphate	HMDB0000224	4.723	141.01893	142.02621	C_2_H_8_NO_4_P	− 1.13	1, 5
C681	− 0.003254398	− 6.39909E-05	1.425411	▼	Ophthalmate	HMDB0005765	4.03	289.12702	290.13429	C_11_H_19_N_3_O_6_	− 1.27	4
C718	0.001894981	− 0.003769861	1.426471	►	sn-Glycero-3-phosphocholine	HMDB0000086	4.433	257.10247	258.10975	C_8_H_20_NO_6_P	− 1.36	5
C240	− 0.001778901	0.003479727	1.461257	◄	Sphinganine	HMDB0000269	1.522	301.29768	302.30496	C_18_H_39_NO_2_	− 1.32	1
C633	− 0.003428438	0.001303504	1.230748	◄	Taurine	HMDB0000251	3.832	125.0144	126.0217	C_2_H_7_NO_3_S	− 1.43	2

1: Sphingolipid metabolism; 2: Taurine and hypotaurine metabolism; 3: Nitrogen metabolism; 4: Cysteine and methionine metabolism; 5: Glycerophospholipid metabolism.

From the analysis, PC1 appears to be associated with H_2_O_2_ administration, while PC2 seems to explain more of the variance related to BLUBE. Variables located far to the right on the loadings plot are likely responsible for pushing the IEC-6 cells exposed to H_2_O_2_-induced oxidative stress and H_2_O_2_-induced oxidative stress in BLUBE-IEC-6 cells away from the other conditions along PC1, such as C123, C749 and C718.

Similarly, variables with large positive or negative loadings on PC2 contribute more to separating IEC-6 cells/BLUBE-IEC-6 cells/ H_2_O_2_-induced oxidative stress in BLUBE-IEC-6 cells and IEC-6 cells exposed to H_2_O_2_-induced oxidative stress which are positioned differently along the PC2 axis in the scores plot, such as C671 and C733 (Table 3).

The complete set of loadings values for PC1 and PC2, along with detailed metabolites information and the group most influenced by each variable, is provided in Table S1 (Supplementary File 1).

#### Partial least squares discriminant analysis (PLS-DA) classification model

In order to assess the specific patterns of BLUBE’s effect on IEC-6 cells and the oxidative stress induced by H_2_O_2_ in IEC-6 cells, a PLS-DA classification model was developed and validated. Given the sample size, a combination of repeated double cross-validation (rDCV) and permutation testing was used for validation. The rDCV procedure involved dividing the samples into 10 cancellation groups for both loops, repeating this procedure 50 times. In order to generate the distribution of classification metrics under the null hypothesis, permutation tests with 1000 randomizations were applied, ensuring that the results were not driven by random correlations. In these tests, class labels were randomly reassigned to the samples, breaking their original groupings, and classification models were constructed based on these permuted labels. The results represented the expected distribution when no real differences existed between categories^45^.

The rDCV PLS-DA modelling strategy provided not only a single estimate of the correct classification rates for individual classes and the whole dataset but also their confidence intervals. Due to the nature of the rDCV procedure, the parameters estimated from the outer loop samples provide a less biased estimate of performance on new, unknown samples. Specifically, in the outer loop, the mean classification accuracy was 98.50 ± 2.31%, indicating the absence of overfitting.

Sensitivity and specificity for each class are reported in Table 4. Sensitivity reflects the model’s ability to correctly classify the groups, with high sensitivity observed for both BLUBE treatment alone (97.25 ± 5.23%) and the combined BLUBE + H_2_O_2_ treatment (99.75 ± 1.77%), while control sensitivity was 96.25 ± 5.06%, and for oxidative stress conditions, it was 98.75 ± 3.79%. Specificity, which measures the model’s capacity to correctly identify true negatives, was also high across all groups, with near-perfect values for the control and H_2_O_2_ groups (99.08 ± 1.74%) and robust results for BLUBE alone (99.42 ± 1.69%) and BLUBE + H_2_O_2_ (99.58 ± 1.26%).

**Table 4:** Sensitivity and specificity (with standard deviations) from rDCV of PLS-DA comparing control (CTRL), H_2_O_2_-induced oxidative stress (H_2_O_2_), BLUBE treatment (M), and combined BLUBE-H_2_O_2_ treatment (M + H_2_O_2_) in IEC-6 cells.

	CTRL	M	H_2_O_2_	M + H_2_O_2_
Sensitivity (std) [%]	96.25 (5.06)	97.25 (5.23)	98.75 (3.79)	99.75 (1.77)
Specificity (std) [%]	99.08 (1.74)	99.42 (1.69)	99.92 (0.59)	99.58 (1.26)

The rDCV used 10 cancellation groups, repeated 50 times, and permutation tests with 1000 randomizations to confirm the results were not due to chance.

Table S2 (Supplementary File 1) further highlights the variables important for prediction (VIP) identified most frequently.

Misclassification proportions for each class are depicted in Fig. S4, demonstrating minimal misclassification, confirming the model’s high accuracy and robustness in distinguishing between conditions.

For the untreated IEC-6 cells predictions, the majority were correctly classified as control, with 96.8% correctly identified and only small misclassification rates (≤ 2.5%) attributed to other classes. This suggests similarities between the effects of BLUBE and the BLUBE + H_2_O_2_ in IEC-6 cells. The H_2_O_2_-IEC-6 cells class showed 98.8% accuracy, with 1.2% of samples misclassified as BLUBE + H_2_O_2_, reflecting that BLUBE did not fully restore normal oxidative damage levels. BLUBE treatment alone exhibited an impressive 99.8% classification rate, confirming that the extract shares some properties with control IEC-6 cells while retaining its unique signature. The BLUBE + H_2_O_2_ in IEC-6 class also showed excellent performance, with 99.8% correct classification, with minimal misclassification (0.2%) to H_2_O_2_, again indicating that the extract only partially restored the cells to their control state.

The rDCV process confirms the robustness of these findings. The sensitivity and specificity across the classes strongly support BLUBE’s protective or restorative role in this cellular model. As shown in Fig. S5, the true model (blue bars) consistently outperformed permuted data (red bars), indicating that the model’s ability to differentiate between experimental conditions is not due to random chance. The high sensitivity and specificity in the IEC-6 and BLUBE groups suggest that these conditions are more easily distinguishable. However, the H_2_O_2_ and BLUBE + H_2_O_2_ groups displayed lower sensitivity and specificity compared to the control, likely due to the overlap between oxidative stress induced by H_2_O_2_ and the partial protective effects of BLUBE. Nonetheless, the model’s performance remained significantly better than chance, as demonstrated by the permutation test results.

#### Pathway enrichment analysis

Based on the VIP scores derived from PLS-DA, significant metabolites were selected to perform a pathway enrichment analysis using their HMDB codes. This analysis revealed that the most enriched pathways were primarily related to metabolism, followed by biosynthesis and degradation. Enrichment methods were applied to identify the most relevant metabolic pathways, with pathway impact and adjusted *p-values* [–log_10_(*p* value)] serving as key metrics. Pathway impact combines centrality and enrichment results, where higher values indicate greater pathway relevance.

**Fig. 6:**
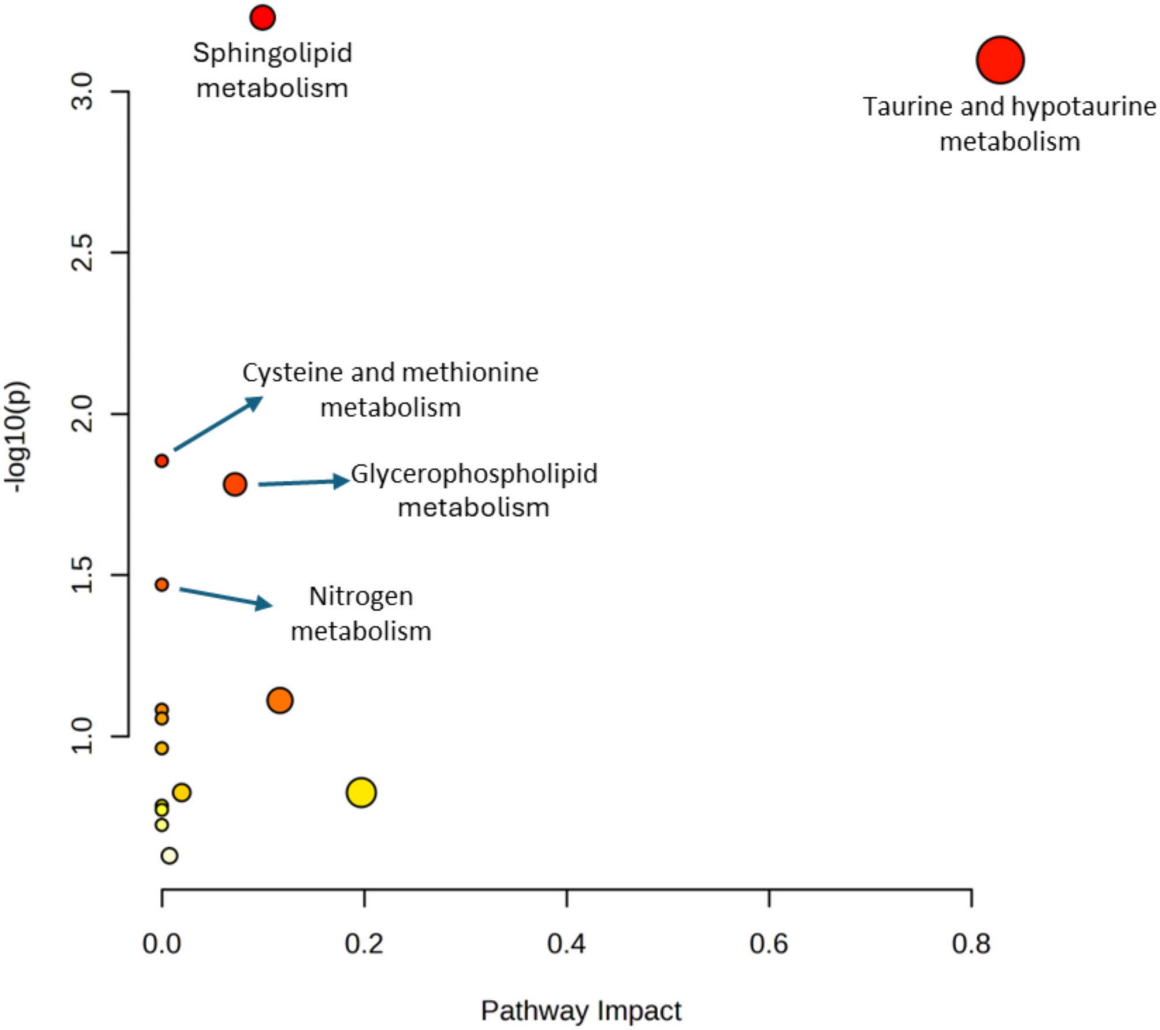
Bubble plot for Kyoto Encyclopedia of Genes and Genomes (KEGG) pathway analysis.

As shown in the bubble chart (Fig. 6), the position of each bubble along the x-axis and its size reflects the pathway’s relative importance, while the y-axis position and color indicate statistical significance, with more intense red shades corresponding to lower *p values*.

The analysis identified that the differentially expressed metabolites detected through untargeted metabolomics were predominantly involved in five significant pathways (*p* value < 0.05): sphingolipid metabolism (*p* = 0.00059), taurine and hypotaurine metabolism (*p* = 0.00079), glycerophospholipid metabolism (*p* = 0.01398), cysteine and methionine metabolism (*p* = 0.01654), and nitrogen metabolism (*p* = 0.03385). The relative abundance of metabolites in these pathways is shown as boxplots in Fig. S6.

## Discussion

Blueberries are increasingly recognized worldwide as healthy food due to their high content of bioactive compounds. They are gaining attention in the pharmaceutical and nutraceutical fields for their antioxidant, anti-inflammatory, anti-proliferative, and potential anticancer properties^21–25^. Their ability to combat oxidative stress, a key factor in the development of various diseases, highlights their cytoprotective potential. Particularly, the use of nutraceutical formulations based on phytochemical extracts, including blueberries, is being explored for their potential in chemoprevention and as adjuvants in supporting pharmacological therapies, primarily due to their ability to mitigate oxidative stress and protect cells from damage at the cellular level^70–74^.

In this work, UHPLC-HRMS characterization allowed for the detection and putative identification of numerous analytes, many of which are generally recognized for their potent antioxidant activity. In particular, various groups of flavonoids, polyphenols and anthocyanins were identified. Among the major flavonoid peaks detected, quercetin hexoside and quercitrin stand out. Both compounds are natural flavonoids found in the flowers, leaves and fruits of various plants and have been widely reported to exhibit anti-inflammatory and antioxidant effects^75^. Additionally, chlorogenic acid, the most abundant compound identified in the extract, along with anthocyanins such as delphinidins, which are characteristically abundant in red fruits, further underscores the potent antioxidant potential of this matrix by effectively scavenging free radicals and mitigating oxidative stress^76^.

The protective mechanism of BLUBE against H_2_O_2_-induced oxidative damage in IEC-6 cells was initially examined through phenotypic assays, confirming the extract’s antioxidant and reparative properties^77,78^.

A healthy and intact intestinal barrier is essential for maintaining gut health and preventing damage^79,80^. Our results show that treatment with H_2_O_2_ impaired the function of the intestinal epithelial barrier. However, the addition of BLUBE significantly reduced hypodiploid nuclei (Fig. 1), and increased mitochondrial activity, wound healing and clonogenic potential (Fig. 2) of IEC-6 cells compared to the H_2_O_2_ group. In addition, a significant reduction in oxidative and nitrosative stress was observed following BLUBE administration in H_2_O_2_-treated cells (Fig. 3).

The actin cytoskeleton is a key regulator of cellular structure and tissue barrier integrity. During oxidative stress, the actin cytoskeleton plays a critical role in regulating cellular integrity and remodelling under both physiological and pathological conditions. In our experiments, we observed that when IEC-6 cells were treated with H_2_O_2_, the actin stress fibers were significantly reduced, and cortical actin density increased. However, treatment with BLUBE restored the normal actin cytoskeleton organization, suggesting its potential to protect and repair the action network under H_2_O_2_ conditions (Fig. 4). Several studies have shown that proper actin filament turnover helps maintain cellular integrity and reduce tissue damage during inflammation, supporting the cytoprotective effects of blueberry extract^81,82^.

Subsequently, its protective efficacy was further validated using cell metabolomics, providing deeper insights into its effects at the metabolic level.

According to metabolic cluster analysis, different pathways associated with oxidative stress are involved, such as taurine, cysteine and glutamate metabolism (Fig. 6).

The loadings and VIP score values of metabolites associated with significant pathways identified in the metabolomic analysis of the different clusters are reported in Table 3.

A dramatic modulation in hypotaurine and taurine content, followed by several other amino acids (L-cystine, L-glutamate) deputed to reduce oxidative stress was found (Fig. S6). Taurine (2-aminoethanesulfonic acid) does not participate in protein synthesis, as it is a non-essential amino acid lacking a carboxyl group. However, it is an important cellular protective agent in the body as osmolyte, keeping a cellular osmotic pressure equal to that of the external fluid environment ensuring protein stability and cell volume under osmotic imbalance^83^. As chemical chaperones, it assists in intracellular folding to stabilize native protein conformations and inhibit aggregation reducing endoplasmic reticulum stress^84–86^. Moreover, it is regarded as a cytoprotective molecule due to its ability to sustain normal electron transport chain being involved in the modification of mitochondrial tRNA for the accurate decoding of certain codons, like UUG, during protein synthesis^87,88^. Additionally, it maintains glutathione stores and upregulate antioxidant responses, increases membrane stability, reduces inflammation and prevents calcium accumulation^89,90^. Increased levels of taurine and hypotaurine in the presence of H_2_O_2_ suggest an adaptive response of intestinal cells to counteract oxidative stress; in this context, BLUBE could amplify this protective effect (Fig. S6g,h).

Accordingly, ophthalmic acid, a metabolite with antioxidant properties, was found significantly modulated in our model (Fig. S6i). Ophthalmic acid is a tripeptide with close structural similarities to GSH, except that it contains 2-aminobutyric acid instead of cysteine. It mimics GSH in some functions and can stimulate certain GSH-dependent reactions while acting as a competitive inhibitor in others. Additionally, it regulates GSH transport by both trans-stimulation and competitive inhibition^91^. The accumulation of ophthalmic acid may indicate an imbalance in GSH synthesis and functions^92,93^. Thus, it plays a significant role in protecting cells from oxidative stress-induced damage scavenging ROS and regulating cellular redox balance highlights its importance in maintaining cellular health and resilience against oxidative stress-related conditions^93,94^. BLUBE could help restore this balance by reducing the need for compensation through ophthalmate production.

Also, sphingolipid and glycerophospholipid metabolism are involved (Fig. S6a-e). Sphingolipids and glycerophospholipids are essential components of cell membranes and play a crucial role in cell signaling, growth regulation, and cell death^95,96^. Key molecules identified and significantly modulated include sphinganine, sphingosine 1-phosphate, *N*-acylsphingosine and sn-glycero-3-phosphocholine^97–99^. Sphingosine 1-phosphate is a potent mediator in cell signaling, promoting cell survival and proliferation. Under oxidative stress conditions, Sphingosine 1-phosphate levels can increase to activate cell survival pathways^100–102^. Sphinganine, a precursor of ceramide, can also accumulate in response to oxidative damage, facilitating membrane repair or inducing cell death in severely damaged cells^103^. *N*-acylsphingosine (ceramide) is associated with stress responses and apoptosis induction^104–106^. Its modulation could represent a balance between inducing apoptosis in damaged cells and promoting the survival of cells protected by the blueberry extract. In each case, H_2_O_2_ can cause cellular membrane damage through lipid peroxidation, and cells might respond by modulating sphingolipid and glycerophospholipid metabolism to repair or remodel damaged membranes^107^. BLUBE extract, through its antioxidant action, may protect membranes, reducing the need for such modifications and leading to a more balanced modulation of sphingolipid metabolites.

## Materials and methods

### Reagents and materials

Water suitable for LC/MS, LiChrosolv®, acetonitrile (ACN) hypergrade for LC–MS LiChrosolv®, formic acid for LC–MS LiChropur™, 2,2-Diphenyl-1-picrylhydrazyl (DPPH), 2,2′-azino-bis(3-ethylbenzothiazoline-6-sulphonic acid) (ABTS), Folin Ciocalteu’s reagent, gallic acid, sodium carbonate, 2,4,6-Trippyridyl-s-triazine (TPTZ), sodium acetate, acetic acid glacial, hydrochloric acid 37%, Iron(III) chloride hexahydrate, Iron(II) sulfate heptahydrate, 3-[4,5-dimethylthiazol-2,5-diphenyl-2H-tetrazolium bromide (MTT), 6-carboxy-2’,7’-dichlorodihydrofluorescein diacetate (DCFH-DA), propidium iodide (PI), hydrogen peroxide were purchased from Merck (Milan, Italy). 4-amino-5-methylamino-2′,7′-difluorofluorescein diacetate (DAF-FM Diacetate) was purchased from Thermo Fisher Scientific (Waltham, Massachusetts, US). Strata-X 33 µm Polymeric Reversed Phase (500 mg 6 mL^−1^) cartridges were purchased from Phenomenex (Bologna, Italy).

### Phytochemical profiling

#### Sample preparation

*Vaccinium myrtillus L.* juice was kindly donated by Vincenzo Montella (La Doria S.p.A., Salerno, Italy) and full data of the used cultivar are reported in Supplementary Material. 10 mL of *Vaccinium myrtillus L.* juice were centrifuged at 6000 rpm for 15 min at 4 °C (Mikro 220R centrifuge, Hettich, Germany) and the supernatants were diluted with H_2_O (1:1 *v*/*v*) and loaded on a Strata™-X 33 µm Polymeric Reversed Phase 500 mg 6 mL^−1^ cartridge.

Solid Phase Extraction (SPE) procedure was applied following Rodriguez-Saona et al.^108^ and Inbaraj et al*.*^109^ protocols, with slight modifications.

After activating the stationary phase with methanol (6 mL) and conditioning it with water (6 mL), the sample was loaded and washed with a water/methanol mixture (95:5, *v:v*, 6 mL) to remove sugars. The polyphenolic components were subsequently eluted using 6 mL of methanol containing 2% formic acid (HCOOH).

The polyphenolic fraction was collected after elution, and the solvent was removed under vacuum evaporation. The resulting blueberry extract (BLUBE) was then stored in a refrigerator at 4 °C until analysis. For subsequent analysis, BLUBE was reconstituted to a concentration of 10 mg mL⁻^1^ in a 50:50 (*v/v*) methanol/water solution containing 0.1% formic acid.

#### Determination of total phenolic content (TPC) and total flavonoids content (TFC)

The TPC of the BLUBE was determined using the Folin–Ciocalteau method as described by Way et al*.*, with slight modifications^110^. 2 μL of BLUBE was added to 100 μL of Folin–Ciocalteu reagent in a microplate, mixed, and left for 5 min before adding 70 μL of sodium carbonate. Then, the microplate was incubated for 1 h at 40 °C. The absorbance of the solution was then evaluated at 765 nm using a microplate reader (Multiskan Go, Thermo Scientific, Waltham, MA, USA). Gallic acid was selected as the standard, the stock solution (1 mg/mL) was prepared in MeOH, and the calibration curve was obtained in a concentration range of 30–500 μg/mL (y = 0.0015x − 0.149; R^2^ = 99.96%). The analysis was performed in triplicate. The total phenolic content was expressed as milligrams of gallic acid equivalents per gram of dry weight (mg GAE g^−1^ dw).

The TFC was determined using the method reported by Imeneo, V. et al., with slight modifications.^111^. An aliquot of extract (25 μL), 100 μL of deionized water, and 7.5 μL of NaNO_2_ (5%, *w/v*) were placed in a microplate and incubated at room temperature (5 min). Then, 7.5 μL of AlCl_3_ (10%, *w/v*) were added and incubated for 6 min; after that, 100 μL of NaOH (4%, *w/v*) was mixed. The reaction mixture was allowed to incubate in the dark for 15 min. After this period, the absorbance was measured at 510 nm using a microplate reader (Multiskan Go, Thermo Scientific, Waltham, MA, USA). The absorbance values were then compared to a rutin calibration standard curve (30–500 μg/mL; y = 0.0005x – 0.0584; R^2^ = 99.97%). The analysis was performed in triplicate. The TFC was expressed as milligrams of rutin equivalents per gram of dry weight (mg RE g^−1^ dw).

#### UHPLC-PDA-ESI-orbitrap-MS/MS conditions

UHPLC-HRMS/MS analysis was performed on a Thermo Scientific™ Vanquish™ UHPLC system, equipped with a VF-P10-A binary solvent delivery system, a VC-D11-A photodiode array detector, a VH-C10-A column compartment and VF-A10-A autosampler. The UHPLC system was coupled online to a Orbitrap Exploris™ 120 mass spectrometer (Thermo Fisher Scientific, Bremen, Germany) equipped with a heated electrospray ionization probe (HESI II) operating in both negative and positive modes.

The chromatographic separation was performed on a Kinetex® EVO C18 column (2.6 µm particle size, pore size 100 Å, L × I.D. 150 × 2.1 mm, Phenomenex, Bologna, Italy). The column temperature and the flow rate were set at 40 °C and 0.4 mL/min, respectively. The mobile phases were: H_2_O (A) and ACN (B) both acidified with 0.5% HCOOH (*v/v*) with the following gradient: 0.01–37.00 min, 3–21% B; 37.01–40.00 min, 21–95% B; 40.01–42.00 min, isocratic to 95% B; 42.01–45.00 min, 95–3% B; then five minutes for column re-equilibration.

The following PDA parameters were applied: sampling rate, 20.0 Hz; detector response time, 0.200 s; and detector peak width, 0.020 min. Data acquisition was set in the range 190–800 nm, and chromatograms were monitored at 280, 330 and 520 nm at maximum absorbance of the compounds of interest.

The MS was calibrated by Thermo Pierce™ FlexMix™ Calibration Solutions in both polarities. Full MS (100–1400 m*/z*) and data-dependent MS/MS were performed at a resolution of 60,000 and 15,000 FWHM respectively; Normalized Collision Energy (NCE) value of 30 was used. Source parameters: Sheath gas pressure, 30 arbitrary units; auxiliary gas flow, 10 arbitrary units; spray voltage, + 3.0 kV, -2.0 kV; capillary temperature, 350 °C; auxiliary gas heater temperature, 300 °C. The identification of investigated analytes was carried out by comparing their retention times and MS/MS data with those present in the literature. Data analysis and processing were performed using FreeStyle™ 1.8 SP2 and the commercial software Compound Discoverer v. 3.3.1.111 SP1 (Thermo Fisher Scientific, Bremen, Germany).

#### Semi-quantitative analysis

For the semi-quantitative analysis of phenolic acids, flavonoids, and anthocyanins, chlorogenic acid and quercetin were used as external standards in negative ionization mode, while cyanidin-3-glucoside was used in positive ionization mode. Stock solutions (1 mg mL^−1^) were prepared in MeOH, and the calibration curves were obtained in a concentration range of 0.1–200 µg mL^−1^ for cyanidin-3-glucoside (CynGlu), 5–750 µg mL^−1^ for chlorogenic acid and 1–500 µg mL^−1^ for quercetin, using five concentration levels with triplicate injections for each level. Linear regression was used to generate the calibration curves with R^2^ values ≥ 0.999. Extracted ion chromatograms (XIC) areas of the standard were plotted against corresponding concentrations (µg mL^−1^). The compound content in the sample was expressed as milligram equivalent per gram of dried extract mean ± deviation standard (n = 3). Limits of detection (LOD) and quantification (LOQ) were calculated by using the standard deviation (SD) and the slope of the calibration curve, multiplied by 3.3 and 10, respectively. The LOD values for chlorogenic acid, quercetin, and cyanidin-3-glucoside were 0.36 μg mL⁻^1^, 0.07 μg mL⁻^1^, and 0.05 μg mL⁻^1^, respectively. The LOQ values for chlorogenic acid, quercetin, and cyanidin-3-glucoside were 1.09 μg mL⁻^1^, 0.22 μg mL⁻^1^, and 0.14 μg mL⁻^1^, respectively.

### In vitro cell-free antioxidant capacity assays

#### DPPH (2,2-diphenyl-1-picrylhydrazyl) test

The free radical scavenging ability of the BLUBE was tested using DPPH radical scavenging assay. The DPPH test was performed with slight modifications to the conditions reported by Noreen et al*.*^112^. Briefly, 20 μL of extract (25–200 μg/mL) or methanol as blank were mixed with 180 μL methanolic solution of DPPH (0.1 mM). BLUBE was prepared in triplicate, shaked and incubated in dark for 30 min at 37 °C. Changes in the absorbance were measured at 517 nm using a microplate reader (Multiskan Go, Thermo Scientific, Waltham, MA, USA), with methanol used as the blank. The DPPH radical scavenging activity of the sample was expressed as Trolox equivalent antioxidant capacity calculated as follows: TEAC = *IC*50*Trolox*/*IC*50*sampl*e. The higher TEAC value means a higher DPPH radical scavenging activity.

#### ABTS (2,2-azinobis (3-ethylbenzothiazoline-6-sulfonate)) test

The ABTS assay was performed according to the method described by Walker and Everette^113^ with modifications. The ABTS solution was prepared with 38.4 mg of ABTS to which was added 6.62 mg of potassium persulphate (K_2_S_2_O_8_) and finally 10 mL of MilliQ water. The mixture was kept in the dark for 16 h. The ABTS solution was diluted with EtOH to obtain an optimal absorbance ranging from 1.20 to 1.30 at 734 nm using a conventional 1 cm spectrophotometer. Subsequently, 2.5 µL of BLUBE (25–200 μg/mL) or EtOH as blank were added to the 96-well microplate, followed by the addition of 250 µL of ABTS solution. The plates were incubated in the dark for 20 min at room temperature and recorded using a microplate reader (Multiskan Go, Thermo Scientific, Waltham, MA, USA). The ABTS radical scavenging activity of the sample was expressed as Trolox equivalent antioxidant capacity.

#### Ferric reducing antioxidant power (FRAP) test

The assay was conducted under the conditions previously described by Aquino et al*.*^114^. Trolox was used as reference (1–200 μg/mL; y = 0.0202x + 0.1323; R^2^ = 99.99%). BLUBE was prepared in triplicate, shaked and incubated in dark for 30 min at 37 °C. The assay is based on the reduction of ferric-tripyridyltriazine (Fe^3+^-TPTZ) to an intense blue color ferrous-tripyridyltriazine complex (Fe^2+^-TPTZ). Changes in the absorbance were measured against blank at 593 nm using a microplate reader (Multiskan Go, Thermo Scientific, Waltham, MA, USA). FRAP activity was calculated as milligrams of trolox equivalents per gram of dry weight (mg TXE g^−1^ dw).

#### Metal binding studies

The metal binding studies were performed as described by Umar et al*.*^115^. The UV absorption of the BLUBE (50 μg/mL) alone or in the presence of CuSO_4_, FeSO_4_ or ZnCl_2_ (40 μM) for 30 min in 20% (*v/v*) ethanol/ buffer (20 mM HEPES, 150 mM NaCl, pH 7.4) was recorded using a microplate reader (Multiskan Go, Thermo Scientific, Waltham, MA, USA) with wavelength ranging from 270 to 400 nm. The final volume of reaction mixture was 1 mL.

### Cellular antioxidant assay

#### Cell cultures and drug treatment

IEC-6 cells (CRL-1592) were obtained from American Type Culture Collection (ATCC, Rockville, MD, USA). These cells, derived from normal rat intestinal crypt cells, were grown in Dulbecco’s Modified Eagle Medium (DMEM, 4500 mg/mL glucose) supplemented with 10% (*v*/*v*) fetal bovine serum, 2 mM L-glutamine, 100 U/mL penicillin, 0.1 mg/mL streptomycin and 0.1 U/mL human insulin. Cells were routinely grown in culture dishes (Corning, Corning, NY) in an environment containing 5% CO_2_ at 37 °C. They were subcultured every 2 days with growth and viability monitored using phase-contrast microscopy and trypan blue staining^116^. In each experiment, cells were placed in a fresh medium, treated with the BLUBE and in the presence of H_2_O_2_ for different experimental times. Each treatment and analysis were performed at least in triplicate separate experiments.

#### Cell viability assay

Cell viability was established by measuring mitochondrial metabolic activity with 3-[4,5-dimethylthiazol-2,5-diphenyl-2H-tetrazolium bromide (MTT)^117^. Briefly, IEC-6 (25 × 10^3^ cells/well) was plated into 96-well plates, then the BLUBE (6.25–200 µg/mL) was added in co-administration with H_2_O_2_ (300 µM) for 24 h. Afterward, MTT reagent at 0.5 mg/mL final concentration for 2 h was added. Then, 100 μL per well of 0.1 M isopropanol/HCl solution was added to dissolve the formazan crystals. The absorbance was measured at 570 nm, using a microplate reader (Multiskan Go, Thermo Scientific, Waltham, MA, USA). Cell viability was expressed as a percentage relative to the untreated cells cultured in medium with 0.1% DMSO and set to 100%, whereas 10% DMSO was used as positive control and set to 0% of viability.

#### ROS and RNS detection

Reactive oxygen species (ROS) and reactive nitrogen species (RNS) levels were measured using respectively 10 μM 6-carboxy-2′,7′-dichlorodihydrofluorescein diacetate (DCFH-DA, Sigma Aldrich, St. Louis, MO, USA) and 5 μM 4-amino-5-methylamino-2′,7′-difluorofluorescein diacetate (DAF-FM-DA, Thermo Fisher Scientific, Waltham, MA, USA)^118^. To test the effect of the BLUBE (50 µg/mL) to ROS and RNS neutralization, IEC-6 cells were seeded (15 × 10^4^ cells/well) in 24-well plates allowing them to adhere for 24 h. Next, cells were incubated for 6 h with BLUBE (50 µg/mL) in co-administration with H_2_O_2_ (300 µM). After 6 h, the medium was removed, and the cells were washed twice with PBS. A staining solution containing DCFH-DA or DAF-FM in serum-free medium without phenol-red was added for 20 min at 37 °C in the dark. The fluorescence signals were evaluated using Becton Dickinson FACScan flow cytometer and analyzed with Cell Quest software, version 4 (Franklin Lakes, NJ, USA).

#### Determination of hypodiploid nuclei

Hypodiploid nuclei were analyzed using propidium iodide (PI) staining by flow cytometry as described previously^119^. IEC-6 cells (15 × 10^4^ cells/well) were grown in 24-well plates and allowed to adhere for 24 h. Later the medium was replaced, and cells were treated with BE (50 µg/mL) in co-administration with H_2_O_2_ (300 µM) for 24 h. After treatments, the culture medium was replaced, cells washed twice with PBS and then suspended in 300 μL of a hypotonic staining solution containing 50 µg/mL PI, 0.1% (*w*/*v*) sodium citrate, and 0.1% Triton X-100. Culture medium and PBS were centrifuged, and cell pellets were pooled with cell suspension to retain both dead and living cells for analysis. After incubation at 4 °C for 30 min in the dark, cell nuclei were analyzed with a Becton Dickinson FACScan flow cytometer using the Cell Quest software, version 4 (Franklin Lakes, NJ, USA). Cellular debris was excluded from the analysis by raising the forward scatter threshold, then the percentage of cells in the hypodiploid region (sub G0/G1) was calculated.

#### Confocal microscopy imaging

IEC-6 cells were seeded (10 × 10^4^ cells/well) in 24-well plates allowing to adhere for 24 h. BLUBE (50 μg/mL) was incubated for 4 h with H_2_O_2_ (300 µM). Cells seeded on glass coverslips were washed in PBS, fixed in PBS-4% paraformaldehyde and permeabilized 5 min in PBS containing 0.1% triton. Then, cells were incubated with a blocking solution containing PBS-0.5% BSA and 50 mM NH_4_Cl for 30 min^120^. The F-actin staining was conducted with phalloidin-tetra-methyl-rhodamine B isothiocyanate (Merk, Darmstadt, Germany) for 30 min (50 µg/mL). Nuclei were counterstained with 1.6 μM Hoechst 33,342 (Sigma Aldrich, St. Louis, MO, USA) during phalloidin staining. Images were acquired on a laser scanning confocal microscope (TCS SP8; Leica MicroSystems) equipped with a plan Apo 63X, NA 1.4 oil immersion objective lens.

#### Colony formation assay

The clonogenic potential was assessed using a subtoxic dose (50 μM) of H_2_O_2_ and BLUBE (50 μg/mL). Cells were plated in 6-well plates at a seeding density of 3 × 10^4^ cells/well. After incubation for 24 h, the culture was terminated by removing the medium and washing the colonies twice with PBS. The cells were fixed and stained with a solution containing 3.7% formaldehyde and 0.5% crystal violet for 30 min, and then washed twice with PBS^121^. Images were obtained and the number of colonies was counted with the free image-processing software ImageJ, version 1.47.

#### Wound healing assay

In wound healing analysis, 1 × 10^6^ cells were seeded in 6-well plates and then incubated at 37 °C for 24 h. After that, a linear scratch was created with a 10 μL sterile pipette tip. Cells were washed twice with PBS and cultured in a medium containing H_2_O_2_ (50 μM), BLUBE (50 μg/mL) or their coadministration. To avoid cell proliferation, DMEM with 1% FBS was used^121^. Each scratch area was photographed at 0 and 24 h. Images were obtained and the wound size for the different times was calculated with the free image-processing software ImageJ, version 1.47.

#### Statistical analysis

Data are reported as mean ± SD of results from three independent experiments. Statistical analysis was performed using an analysis of variance test (ANOVA), and multiple comparisons were made with Bonferroni’s test with GraphPad Prism 8.0 software (San Diego, CA, USA). Significance was assumed at *p* < 0.05.

### Metabolomics

#### Sample preparation

IEC-6 cells were seeded (3 × 10^6^ cells) in 60 mm plates. After 24 h, the cells were treated with the BLUBE (50 μg/mL), H_2_O_2_ (150 µM) and in their combination for 8 h. Subsequently, the medium was removed, and the cells were washed twice with PBS, then detached using 80% cold methanol solution. The suspension cells were centrifuged at 1000 g at 4 °C for 10 min. Both the pellet and the supernatant were collected for analysis. Samples were stored at -80 °C until metabolites were extracted.

For metabolite extraction, 500 µL of chilled acetonitrile/methanol/water (2:2:1, *v:v:v*) was added to pellet samples^122^. Protein precipitation was performed by 2 cycles of vortex for 30 s, incubation at − 30 °C for 1 min and sonication for 10 min. This was followed by incubation at − 30 °C for 30 min and centrifugation at 14,000 rpm for 10 min at 4 °C. The supernatants were then transferred to another tube and evaporated to dryness using a SpeedVac (Savant, Thermo Scientific, Milan, Italy). The dry metabolite extracts were reconstituted with 100 µL acetonitrile/water (1:1, *v:v*) and injected for UHPLC-ESI-Orbitrap-MS/MS analysis.

#### UHPLC-ESI-orbitrap-MS/MS conditions

The chromatographic separation was performed on a ACQUITY UPLC BEH Amide column (1.7 µm particle size, pore size 130 Å, L × I.D. 2.1 × 100 mm, Waters Corporation, Milford, MA, USA). The column temperature and the flow rate were set at 45 °C and 0.4 mL/min, respectively. The mobile phases were: H_2_O (A) and ACN (B) both acidified with 0.1% HCOOH (*v/v*) with the following gradient: 0.01–0.10 min, isocratic to 99% B; 0.11–7.00 min, 99–30% B; 7.01–7.50 min, isocratic to 30% B; 7.51–7.60 min, 30–99% B; then three minutes for column re-equilibration.

Data were acquired in positive ionization mode. Full MS (70–800 m*/z*) and data-dependent MS/MS were performed at a resolution of 30,000 and 15,000 FWHM respectively; Normalized Collision Energy (NCE) values of 20, 40, 60 were used. Source parameters: Sheath gas pressure, 40 arbitrary units; auxiliary gas flow, 15 arbitrary units; spray voltage, + 3.4 kV, − 2.0 kV; capillary temperature, 280 °C; auxiliary gas heater temperature, 300 °C.

For data analysis, Compound Discoverer 3.3 software (Thermo Fisher Scientific, SanJose, CA, USA) was used for raw data processing (baseline correction, noise filtering, spectral alignment, and peak detection) and for putative identification of metabolites based on molecular formula (matched), exact mass (mass tolerance < 5 ppm) and MS^2^ fragmentation pattern [Fragment Ion Search (FISh)], with a global database search (mzCloud, MassList and ChemSpider).

#### Pathway analysis

Metabolic pathway analysis was performed with the MetaboAnalyst 6.0 tool (available online at https://www.metaboanalyst.ca/MetaboAnalyst/). Differentially expressed metabolites were mapped to metabolic pathways using the Kyoto Encyclopedia of Genes and Genomes (KEGG) database. The significance levels were selected at 0.05 for the *p*-value.

#### Chemometric analysis

The filtered dataset was processed and analysed using MATLAB R2023a (The MathWorks Inc., Natick, MA, USA). A combination of custom-developed routines and built-in functions were employed to perform all computational tasks.

#### Pre-processing data

A comprehensive preprocessing pipeline was implemented to prepare the data for subsequent statistical and chemometric analyses while minimizing the impact of noise and systematic variation. Probabilistic Quotient Normalization (PQN)^123^ was applied to the metabolite data to address systematic biases across samples. Each sample was scaled relative to the median value of all control samples, thereby reducing variability associated with sample-specific differences. Logarithmic transformation (base 10) was then applied to stabilize variance and reduce the influence of large values, ensuring a more uniform distribution of the data. Prior to chemometric modeling, autoscaling was performed, centering each variable by subtracting its mean and scaling by dividing by its standard deviation. This ensured that all variables contributed equally to the analysis, improving the performance of multivariate statistical techniques. Details of the preprocessing steps are provided in the Supplementary Material (*SM1 Pre-processing Data*).

Both exploratory (unsupervised) and predictive (supervised) multivariate analyses were performed. Principal Component Analysis (PCA) was conducted to identify patterns and reduce experimental variability, while Partial Least Squares-Discriminant Analysis (PLS-DA) was used to model relationships between the metabolic profiles and experimental conditions, facilitating the identification of significant features.

#### Exploratory tools: principal component analysis (PCA) and data reconstruction

Following column autoscaling to standardize the dataset, Principal Component Analysis (PCA) was performed^124^. To evaluate PCA’s effectiveness in differentiating sample groups, Hotelling’s (T^2^) confidence ellipses were added to the score plots at a 95% confidence level, visually assessing group separation. Additionally, initial PCA analyses using technical replicates and Quality Control (QC) samples were performed to investigate sources of experimental variability^125^. Based on these analyses, PCs associated with experimental noise, such as batch effects, were excluded from further analyses. The dataset was then reconstructed using only biologically relevant PCs, retaining meaningful biological variability while minimizing the influence of experimental artifacts. This reconstructed dataset facilitated unbiased averaging of technical replicates and enabled subsequent exploratory and predictive analyses to focus on meaningful biological patterns^125^.

More information is provided in the Supplementary Material (*SM2 Exploratory tools: Principal Component Analysis (PCA) and Data Reconstruction*).

#### Chemometric classification models

To classify the four distinct groups, we employed PLS-DA, a widely used supervised classification technique. The number of Latent Variables (LVs) used in the model was optimized through a five-fold cross-validation procedure (Venetian blinds) to reduce misclassification errors and enhance accuracy^124,125^. Once the predictive models were constructed, their robustness and ability to generalize to new data were evaluated using repeated Double Cross-Validation (rDCV)^125^. This method was selected due to the limited sample size, ensuring an unbiased validation process^126^. Additionally, to confirm that the model’s performance was not due to chance, permutation tests were conducted, comparing the observed metrics with null distributions obtained through non-parametric methods^127^. Model performance was further evaluated using key metrics: sensitivity, specificity and accuracy^128^. More details are provided in the Supplementary Material (*SM3 Chemometric classification models*).

## Conclusions

By modulating oxidative stress responses and restoring metabolic homeostasis, BLUBE demonstrated its protective potential against oxidative damage, suggesting its therapeutic promise for managing intestinal disorders. The results of this study support the antioxidant and anti-inflammatory effects of the blueberry while also demonstrating the value of HR-MS-based metabolomics in biochemical research. HR-MS metabolomics can significantly contribute to the study of the mechanisms of action of nutraceuticals and drugs by enabling detailed characterization of treatment-induced metabolic changes at the cellular level. This type of approach is particularly suitable in the case of natural compounds, which possess multiple and often unidentified intracellular targets.

The observed metabolic effects, combined with BLUBE cytoprotective and wound-healing properties, underscore its potential as a potent nutraceutical for treating oxidative stress-related intestinal disorders.

## Electronic supplementary material

Below is the link to the electronic supplementary material.

Supplementary Material 1

Supplementary Material 2

Supplementary Material 3

## Data Availability

Data is provided within the manuscript or supplementary information files.
