# Supplementary material for: Metabolomics insights into the protective molecular mechanism of Vaccinium myrtillus against oxidative stress in intestinal cells
Source: Sci Rep. 2025 Mar 13;15:8643. doi: 10.1038/s41598-025-93722-x (PMC11906781; doi:10.1038/s41598-025-93722-x)
Supplement: Supplementary file 2 — Supplementary Material 2 [file 41598_2025_93722_MOESM2_ESM.pdf]

LaDoria GROUP	**M07PQ08_16.10.2023 SPECIFICA TECNICA /** *TECHNICAL SPECIFICATION*	
	Codice prodotto/*Product* code: **16260091**	Descrizione prodotto /*Description product:* ***Purea di mirtilli in fusti asettici*** / *Blueberry puree in aseptic drums*
	CATEGORIA/*Category* **Semilavorati di frutta** *Fruit derivates*	Rev. 0 Data: 16.10.2023 Pag. **1** a **13**

SEZIONE 1<br>SECTION 1	CARATTERISTICHE GENERALI<br>GENERAL CHARACTERISTICS
SEZIONE 2<br>SECTION 2	SPECIFICHE PRODOTTO<br>PRODUCT SPECIFICATION
SEZIONE 3<br>SECTION 3	ALLERGENI, INTOLLERANZE, DERIVATI ANIMALI<br>FOOD ALLERGEN, INTOLLERANCE, ANIMAL PRODUCT
SEZIONE 4<br>SECTION 4	CONTAMINANTI E CORPI ESTRANEI<br>CONTAMINANTS AND FOREIGN BODIES
SEZIONE 5<br>SECTION 5	OGM, CATENA DI FORNITURA E RADIAZIONI IONIZZANTI<br>GMO, SUPPLYCHAIN AND IONIZED RADIATION
SEZIONE 6<br>SECTION 6	SHELF-LIFE E CONFEZIONAMENTO<br>SHELF LIFE AND PACKAGING INFORMATION
SEZIONE 7<br>SECTION 7	TRASPORTO<br>TRANSPORT
SEZIONE 8<br>SECTION 8	NOTE-SCHEDA DI SICUREZZA E RIFERIMENTI LEGISLATIVI<br>NOTES- SAFETY DATA SHEET- LEGISLATION
SEZIONE 9<br>SECTION 9	REVISIONI STORICHE<br>HISTORIC RELEASE
SEZIONE 10<br>SECTION 10	LISTA FORNITORI<br>SUPPLIER LIST

**DATE - FIRMA / SIGNATURE**

SEZIONE 1 - CARATTERISTICHE GENERALI
SECTION 1 - GENERAL CHARACTERISTICS

1.a ***DENOMINAZIONE LEGALE PRODOTTO*** *LEGAL DENOMINATION*

Purea di mirtillo nero
Blueberry puree

1.b ***DESCRIZIONE*** *DESCRIPTION*

Purea di mirtillo nero, con sapore e odore caratteristico del frutto maturo. Purea ottenuta da mirtilli selvatici e/o coltivati sani (Vaccinium Myrtillus), puliti e maturi con setaccio da 0,8mm.

Blueberry puree, with characteristic taste and flavour of ripe fruit. Puree obtained from healthy, clean, and completely ripe wild blueberry and or cultivated (Vaccinium Myrtillus), sieved 0,8mm.

1.c COMPOSIZIONE E ORIGINI/PAESI DI PROVENIENZA
INGREDIENTS COMPOSITION AND ORIGIN

**Ingrediente e sottocomponente** *Ingredients and subcomponent*	**%**	**Origine *** *Country of Origin*	**Paese di Produzione*** *Country of manufacturing*	**° brix**
**Purea di mirtillo nero** *Blueberry*	**100**	**Bielorussia, Canada, Cile, Finlandia, Paesi Baltici, Perù, Polonia, Portogallo, Svezia, Ucraina** *Belarus, Canada, Chile, Finland, Baltic States, Peru, Poland, Portugal, Sweden, Ukraine*		**9,0 – 12,5**

* Indicare il paese di origine e paese di produzione
*Please, indicate country of origin and manufacturing

1.d DICHIARAZIONE IN ETICHETTA
LABEL DECLARATION

Purea di mirtillo nero / Blueberry puree (100%)

DATE - FIRMA / SIGNATURE

SEZIONE 2 - SPECIFICHE PRODOTTO
SECTION 2 - PRODUCT SPECIFICATION

2.a CARATTERISTICHE ORGANOLETTICHE E SENSORIALI *QUALITY STANDARD*

PARAMETRO *PARAMETER*	TARGET-TOLLERANZA *TARGET-TOLERANCE*	FOTO PRODOTTO STD *Picture*
COLORE *COLOUR*	Viola scuro *Dark Purple*	
SAPORE *TASTE*	Tipico mirtillo, nessuna nota bruciata o spenta *Typical blueberry, no burned or off notes*	
ASPETTO *APPEARANCE*	Liquido viscoso purea, senza semi *Viscous liquid puree, seedless*	

2.b CARATTERISTICHE CHIMICO FISICHE
CHEMICAL PHYSICAL CHARACTERISTICS

**PARAMETRO PARAMETER**	**U.M.**	**TARGET- TOLLERANZA TARGET -TOLERANCE**
**Brix rifrattometrico (20 °C)** *Brix refractometric (20 °C)*	°brix	9,0 – 12,5
**Acidità totale (g. ac. citrico anidro/100 g)** *Total acidity (g. anhyd. citric acid. /100 g)*	g/100g	0,7 – 2,0
**pH**		2,70 – 3,70
**Consistenza Bostwick (cm/30”)** *Bostwick texture (cm/30 sec)*	cm	9,0 – 15,0
**Polpa** *Pulp*	%	40 - 60
**Raffinazione (grandezza setaccio)** *Refining (mesh size)*	mm	0,8

2.c CARATTERISTICHE MICROBIOLOGICHE
MICROBIOLOGICAL CHARACTERISTICS

**PARAMETRO** PARAMETER	**U.M.**	***VALORI E TOLLERANZA*** *VALUES AND TOLERANCE*
Carica microbica totale/*Total count*	ufc/g	< 100
Lieviti/*Yeasts*	ufc/g	< 10
Muffe/*Moulds*	ufc/g	< 10
E. coli	ufc/g	< 10
Staphyloccoccus aureus	ufc/g	< 10
Salmonella	ufc/25g	Assente - Absent
Listeria monocytogenes	ufc/25g	Assente - Absent

DATE - FIRMA / SIGNATURE

2.d ANALISI NUTRIZIONALE (valori medi su 100g di prodotto)
NUTRITIONAL ANALYSIS (average value for 100g of product)

**Parametro /** *Parameter*	**U.M.**	**valore /** value
Energia / *energy*	Kcal/Kjoule	47/197
carboidrati / *carboydrates*	g	8,5
di cui zuccheri /*of whic sugar*	g	8,5
grassi / *fat*	g	0,5
di cui saturi /*of which satured*	g	xx
di cui TRANS /*of which trans*	g	xx
proteine / *proteins*	g	0,4
fibra / fiber	g	1,7
sale (NaCl) / *salt*	mg	0,003

2.e CARATTERISTICHE MERCEOLOGICHE / DIFETTOSITA'
PRODUCT CHARACTERISTICS / DEFECTS

**DIFETTOSITA'** DEFECTS	**TOLLERANZA** TOLERANCE	**note**
**n.a**		

**DATE - FIRMA / SIGNATURE**

SEZIONE 3 PRODUTTORE*/SECTION 3 PRODUCER*
ALLERGENI*, INTOLLERANZE, DERIVATI ANIMALI
FOOD ALLERGEN, INTOLLERANCE, ANIMAL PRODUCT*

INGREDIENT CATEGORY	CONTAINS YES/NO	Present on the production line YES/NO	Present on site YES/NO	MAY CONTAIN YES/NO	NOTE
Cereals containing gluten and products thereof					
Crustaceans and products thereof					
Eggs and products thereof					
Fish and products thereof					
Peanuts and products thereof					
Soybeans and products thereof					
Milk and products thereof (including lactose)					
Nuts and products thereof					
Celery and products thereof					
Mustard and products thereof					
Sesame seeds and products thereof					
Sulphur dioxide and sulphites (concentration > 10 ppm)					
Lupine and products thereof					
Molluscs and products thereof					
Meat and product from animal					Indicate species:
Honey					
Shellac					
palm oil or derivated by					Certificate RSPO (mass balance, segregated, IP)
**processing aid, enzymes, nanomaterials**					specify type:
Animal lecithins, beeswax, lactitol, carmine, cochineal and carminic acid, mono and diglycerides of animal fatty acids, vitamin D3 derived from animals, fruits and citrus fruits coated with animal wax					

**DATE - FIRMA / SIGNATURE**

SEZIONE 3 COMMERCIANTE/*SECTION 3 TRADER*

☐ **solo commercializzazione/*only warehouse -no handling***	☐ **manipolazione, confezionamento/*handling and packing***

ALLERGENI*, INTOLLERANZE, DERIVATI ANIMALI
FOOD ALLERGEN, INTOLLERANCE, ANIMAL PRODUCT*

**INGREDIENT CATEGORY**	**CONTAINS**	**Present on the production line**	**Present on site**	**MAY CONTAIN**	**NOTE**
Cereals containing gluten and products thereof					
Crustaceans and products thereof					
Eggs and products thereof					
Fish and products thereof					
Peanuts and products thereof					
Soybeans and products thereof					
Milk and products thereof (including lactose)					
Nuts and products thereof					
Celery and products thereof					
Mustard and products thereof					
Sesame seeds and products thereof					
Sulphur dioxide and sulphites (concentration > 10 ppm)					
Lupine and products thereof					
Molluscs and products thereof					
Meat and product from animal					Indicate species:
Honey					
Shellac					
palm oil or derivated by					Certificate RSPO (mass balance, segregated, IP)
**processing aid, enzymes, nanomaterials**					specify tyoe:
Animal lecithins, beeswax, lactitol, carmine, cochineal and carminic acid, mono and diglycerides of animal fatty acids, vitamin D3 derived from animals, fruits and citrus fruits coated with animal wax					

DATE - FIRMA / SIGNATURE

SEZIONE 4 - CONTAMINANTI E CORPI ESTRANEI
SECTION 4 - CONTAMINANTS AND FOREIGN BODIES

4.a CONTAMINANTI, TOSSINE, RESIDUI DI PESTICIDI, METALLI PESANTI E/O ALTRE SOSTANZE INDESIDERABILI
CONTAMINANTS, TOXINS, PESTICIDE RESIDUES, HEAVY METALS AND/OR ANY OTHER SUBSTANCE INDESIDERABLE

I fornitori garantiscono che il prodotto fornito rispetta i limiti riportati nel Codex Alimentarius (www.codexalimentarius.net) e nella legislazione europea vigente relativamente alla presenza di contaminanti, tossine, residui di pesticidi, farmaci veterinari, microrganismi ed altre sostanze indesiderabili nel prodotto e nel materiale di confezionamento.
The suppliers guarantees the maximum limits set by the Codex Alimentarius and European Regulations for the presence of contaminants, toxins, pesticide residues, veterinary drugs residues, microorganisms and other undesirable substances in foods and packaging materials.

**Parametro /** *Parameter*	**U.M.**	**Tolleranza /** *Tolerance*	**frequenza /** *frequency*
Metalli pesanti<br>*Heavy metals*	In accordo con il Regolamento UE 2023/915 e successive modifiche<br>*In accordance with EU Regulation 2023/915 and further amendments*		Ogni anno, presso laboratorio esterno<br>Once a year / by External Lab
Pesticidi<br>*Pesticides*	Entro i limiti massimi di residui (LMR) previsti dal Regolamento Europeo n°396/05, n°178/2006, n°149/2008 e successivi<br>*Within maximum residual limits (LMR) provided for by EU regulation no°396/05, no°178/2006, n°149/2008 and further amendments*		
Micotossine: Patulina<br>Micotoxins: Patulin	In accordo con il Regolamento UE 2023/915 e successive modifiche<br>*In accordance with EU Regulation 2023/915 and further amendments*		

4.b CORPI ESTRANEI
FOREIGN MATERIALS

Il fornitore garantisce di aver messo in atto un insieme di procedure, controlli e sistemi di monitoraggio idonei ad eliminare il rischio di presenza di corpi estranei nel prodotto. Per corpo estraneo si intende qualsiasi sostanza che si trovi nel prodotto ma non direttamente designata per far parte di quel prodotto e si classifica come critico, maggiore e minore a seconda della pericolosità.
The supplier guarantees to have implemented any procedure, action, or monitoring system in order to avoid the presence of foreign materials on the finished product. Foreign material is any object or material which may become part of the product, but which is not designed to be a part of such product. This can be critical, major, or minor in function of the hazard for the consumer.

DATE - FIRMA / SIGNATURE

**critico**<br>*critical*	questo corpo estraneo è pericoloso e può comportare un potenziale danno al consumatore<br>*this foreign material is hazardous and can potentially cause harm for the consumer*
**maggiore**<br>*major*	questo corpo estraneo non è pericoloso a bassi livelli e la sua presenza è sgradevole nel prodotto<br>*this foreign material is not hazardous at low levels and the presence is not desiderable in the product*
**minore**<br>*minor*	questo corpo estraneo non è pericoloso a bassi livelli e la sua presenza è sgradevole nel prodotto ma non è tecnicamente eliminabile, pertanto, viene tollerato a bassi livelli<br>*this foreign material is not hazardous at low levels and the presence is not desiderable in the product but is technically unavoidable, so is tolerated at low levels*

**Parametro /** *Parameter*	**U.M.**	**Tolleranza /** *Tolerance*
**critico: Corpi estranei pericolosi (vetro-metallo, plastica ecc..)**<br>*critical: hazardeous foreign material (glass, plastic, metal ecc)*	(w/w)	ASSENTE-ABSENT
**maggiore Corpi estranei vegetali**<br>*major: Vegetable foreign material*	(w/w)	ASSENTE-ABSENT
Minore<br>***Minor***	(w/w)	ASSENTE-ABSENT

DATE - FIRMA / SIGNATURE

SEZIONE 5 - OGM, CATENA DI FORNITURA E RADIAZIONI IONIZZANTI
SECTION 5 - GMO, SUPPLYCHAIN AND IONIZED RADIATION

5.a **I fornitori dichiarano che le materie prime/ingredienti forniti al Gruppo La Doria non contengono né derivano da organismi geneticamente modificati né contengono DNA o proteine derivati da modificazioni genetiche. Il prodotto è conforme alla legislazione REG (EU)1829/2003 e 1830/2003.**

THE SUPPLIERS DECLARE THAT THE RAW MATERIALS/INGREDIENTS SUPPLIED TO LA DORIA GROUP NOT CONTAIN AND ARE NOT DERIVED FROM GMO.
The product is non-GMO according to EU legislation. No labelling according to the Regulations (EC) No 1829/2003 and No 1830/2003 is caused.

5.b **Il fornitore dichiara che il prodotto fornito al Gruppo La Doria non è stato sottoposto a radiazioni ionizzanti, tale dichiarazione si riferisce anche alle singole materie prime contenute.**
THE SUPPLIER DECLARES THAT THE PRODUCT SUPPLIED TO LA DORIA GROUP IS NOT IRRADIATED OR TREATED WITH IONIZING RADIATION. THIS IS REFERRED ALSO TO THE INDIVIDUAL INGREDIENTS AND RAW MATERIALS CONTAINED.

DATE - FIRMA / SIGNATURE

SEZIONE 6 - SHELF-LIFE E CONFEZIONAMENTO
SECTION 6 - SHELF LIFE AND PACKAGING INFORMATION

6.a CONFEZIONAMENTO *PACKAGING*

IMBALLO PRIMARIO *PRIMARY PACKAGING*

**Descrizione materiale di imballaggio** *Description and type of material**	**Peso netto** Product *net weight*	**Temperatura** *temperature*	**Temperatura** *temperature*	**note** *notes*
Busta asettica *Multilayer inliner aseptic bag*	200kg	0 – 5°C		

IMBALLO SECONDARIO *SECONDARY PACKAGING*

**Descrizione** *Description*	**Peso netto** product *net weight*	**Temperatura** *temperature*	**note** *notes*
Fusti metalli *Metal drums*	200 kg	0 – 5°C	

6.b SHELF-LIFE E MODALITA' DI STOCCAGGIO *SHELF LIFE AND STORAGE CONDITIONS*

**Descrizione** *Description*	**UM**	**numero** *number*	**temperatura** *temperature*	**note** *notes*
**SHELF LIFE**	Mesi Months	12	0 – 5°C	Freeze if opened

6.c SHELF LIFE RESIDUA MINIMA E MODALITA' DI STOCCAGGIO ALLA CONSEGNA
MINIMUM *SHELF LIFE FROM THE DELIVERY AND STORAGE CONDITIONS*

**Descrizione** *Description*	**% *SHELF LIFE* TOTALE** *TOTAL*	**UM**	**numero** *number*	**Temperatura** *temperature*
**SHELF-LIFE RESIDUA remaining shelf life**	75	Mesi Months	9	0 – 5°C

6.d INDICAZIONI DI RICONOSCIMENTO/ *CODING AND LABELLING INFORMATION REQUIRED*

CODIFICHE PRESENTI SULL'UNITA' DI CONSEGNA (OLTRE QUELLE DI LEGGE)
CODES TO BE PRESENT ON THE UNIT DELIVERY (BEHOND THE REGULATION REQUIREMENTS)

***Lotto** *Lot/batch code*	***Denominazione legale del prodotto** *Legal denomination of the product*	***data di fabbricazione** *production date*
***Nome del produttore ed indirizzo dello stabilimento di produzione** *Name of the Producer and address of the production site*		***Peso netto** *Net Weight*
***Modalità di conservazione e TMC** *Storage conditions and Shelf life of the product*		***Informazioni Allergeni** *Allergen information*

DATE - FIRMA / SIGNATURE

SEZIONE 7 - TRASPORTO
SECTION 7 - TRANSPORT

INSERIRE LA MODALITA' DI TRASPORTO UTILIZZATA ED EVENTUALI PRESCRIZIONI DA RISPETTARE
INSERT TRANSPORT INFORMATION AND ANY CONSTRAINTS TO BE CONSIDERED

Trasporto in condizioni temperatura controllata (0-5°C). Il vettore deve presentare tracciato termico conforme dal momento del carico fino all'arrivo.

Transport at 0-5°C. The carrier must show the registration of the temperature from the loading time to the arrival, compliant with range 0-5°C.

DATE - FIRMA / SIGNATURE

SEZIONE 8 - NOTE-SCHEDA DI SICUREZZA E RIFERIMENTI LEGISLATIVI
SECTION 8 - NOTES- SAFETY DATA SHEET- LEGISLATION

8.a NOTE: DOCUMENTI RICHIESTI ED EVENTUALI PRESCRIZIONI SPECIFICHE
NOTES: DOCUMENTS REQUIRED AND SPECIFIC PRESCRIPTIONS

- Analisi pesticidi, metalli pesanti e patulina1/anno
Pesticides, heavy metals and patulin analysis1/year

- Certificato analisi radioattività (Cesio radioattivo) 1/anno
Radoiactivity certificate (Cesium radioactive) 1/year

- ***Analisi migrazione materiale di imballaggio 1/2 anni***
Migration packaging material analysis 1/each 2 years

NB: dal momento che il prodotto è usato in ricette free from si richiede sul DDT la presenza della dicitura "prodotto esente da cereali contenenti glutine".
Since the product is used in free from recipe it must be reported on the Delivery document "product free from gluten and cereals containing gluten"

8.b ALLEGATA SCHEDA DI SICUREZZA

ATTACHED SAFETY DATA SHEET

8.c RIFERIMENTI LEGISLATIVI *LEGISLATION*

Reg (CE) 178/2002 - principi e requisiti generali della legislazione alimentare
DL 193/2007 - attuazione della Dir 2004/41/CE relativa ai controlli in materia di sicurezza alimentare
Reg (CE) 2073/2005 - criteri microbiologici applicabili ai prodotti alimentari
Reg (UE) 1169/2011 etichettatura dei prodotti alimentari
Reg (CE) 1333/2008 e successive modifiche -Additivi alimentari
Reg (CE) 1334/2008 e successive modifiche -Aromi
Reg (CE) 1935/2004 - materiali ed oggetti destinati a venire a contatto con i prodotti alimentari
DIR (CE)89/2003 Allergeni- DIR 2007/68/CE che modifica l'Allegato III bis della Direttiva 2000/13/CE
REG (CE) 1829/2003-1830/2003 Organismi geneticamente modificati
Reg (CE) 396/2005 Limiti residui pesticidi negli alimenti
REG (UE) 2023/915 Contaminants
Direttiva 2012/112/CE Fruit juices
AIJN Guidelines

9. REVISIONI STORICHE/ *HISTORIC* ***RELEASE***

**data** *date*	**revisione** *release*	**sezione** *section*	**motivazione** *motivation*
**16.10.2023**	**0**		**Nuovo format /***New format*

DATE - FIRMA / SIGNATURE

10. LISTA FORNITORI /*SUPPLIER LIST*

**Fornitore** *Supplier*	**Data inserimento** *Date*	**Note** *Note*

**DATE - FIRMA / SIGNATURE**
