# Supplementary material for: Metabolomics insights into the protective molecular mechanism of Vaccinium myrtillus against oxidative stress in intestinal cells
Source: Sci Rep. 2025 Mar 13;15:8643. doi: 10.1038/s41598-025-93722-x (PMC11906781; doi:10.1038/s41598-025-93722-x)
Supplement: Supplementary file 3 — Supplementary Material 3 [file 41598_2025_93722_MOESM3_ESM.docx]

**SUPPLEMENTARY MATERIALS**

**Metabolomics insights into the protective molecular mechanism of *Vaccinium myrtillus* against oxidative stress in intestinal cells**

Sara Novi ^1,#^, Vicky Caponigro ^1,#^, Maria Rosaria Miranda ^1,2,5^, Giovanna Aquino ^1,2^, Matteo Delli Carri ^1^, Emanuela Salviati ^2^, Silvia Franceschelli ^1^, Carla Sardo ^1^, Manuela Giovanna Basilicata ^3,*^, Vincenzo Vestuto ^1,*^, Mario Felice Tecce ^1^, Federico Marini ^4^ , Giacomo Pepe ^1,5^, Pietro Campiglia ^1^, and Michele Manfra ^6^

^1^ Department of Pharmacy, University of Salerno, Via G. Paolo II, Fisciano, 84084 Salerno, Italy;

[snovi@unisa.it](mailto:snovi@unisa.it); [vcaponigro@unisa.it](mailto:vcaponigro@unisa.it); [mmiranda@unisa.it](mailto:mmiranda@unisa.it); [gaquino@unisa.it](mailto:gaquino@unisa.it); [mdellicarri@unisa.it](mailto:mdellicarri@unisa.it); [esalviati@unisa.it](mailto:esalviati@unisa.it); [sfranceschelli@unisa.it](mailto:sfranceschelli@unisa.it); [vvestuto@unisa.it](mailto:vvestuto@unisa.it); [tecce@unisa.it](mailto:tecce@unisa.it); [gipepe@unisa.it](mailto:gipepe@unisa.it); [pcampiglia@unisa.it](mailto:pcampiglia@unisa.it).

^2^ PhD Program in Drug Discovery and Development, University of Salerno, Fisciano, 84084 Salerno, Italy.

^3^ Department of Advanced Medical and Surgical Sciences, University of Campania “Luigi Vanvitelli”, Naples, Italy; [manuelagiovanna.basilicata@unicampania.it](mailto:manuelagiovanna.basilicata@unicampania.it)

^4^ Department of Chemistry, University of Rome “La Sapienza”, Piazzale Aldo Moro 5, 00185 Rome, Italy; [federico.marini@uniroma1.it](mailto:federico.marini@uniroma1.it)

^5^ National Biodiversity Future Center (NBFC), 90133 Palermo, Italy

^6^ Department of Science, University of Basilicata, Viale dell’Ateneo Lucano 10, 85100 Potenza, Italy; [michele.manfra@unibas.it](mailto:michele.manfra@unibas.it)

**#** These authors are co-first authors

* Correspondence: [vvestuto@unisa.it](mailto:vvestuto@unisa.it); [manuelagiovanna.basilicata@unicampania.it](mailto:manuelagiovanna.basilicata@unicampania.it)


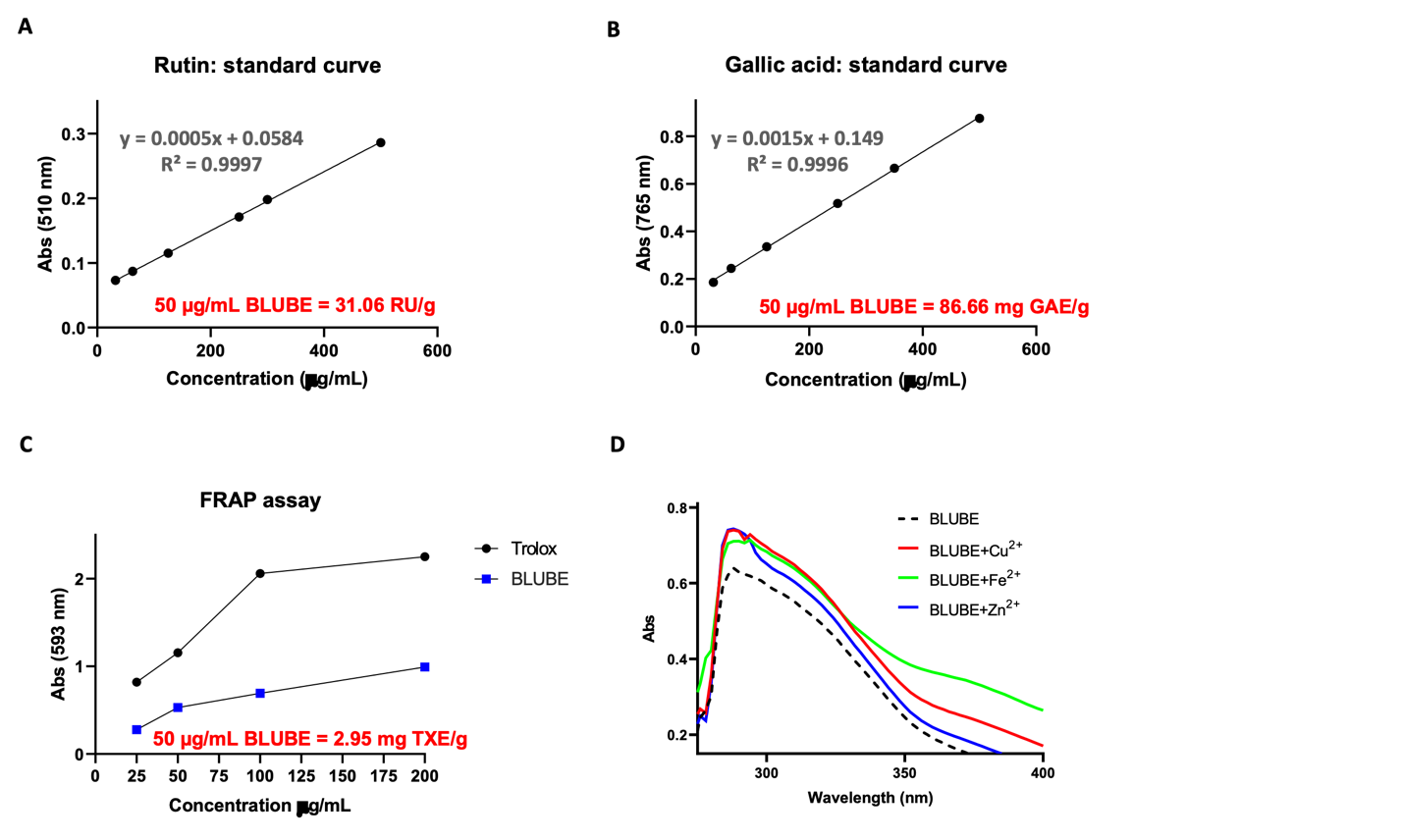


**Figure S1**. In vitro antioxidant properties of BLUBE and its bioactive content. Standard curves of

rutin and gallic acid used respectively for (A) total flavonoid and (B) phenolic content determination. (C) FRAP assay, (D) metal chelating activity. Results are showed as mean ± standard deviation (SD) from three independent experiments.

*SM1 Pre-processing Data*

The preprocessing pipeline began with normalization, which involved calculating the median value for each sample, excluding zeros and missing (NaN) values. A custom function was developed to handle NaNs and zero entries in the dataset. This function replaced such values with random values generated using a scaling factor of 1/5 of the minimum non-zero value in each column of the dataset. This approach ensured the replacement values were small yet consistent with the data's scale, preserving the dataset's overall distribution and variability.

After normalization, logarithmic transformation (base 10) was applied to stabilize variance and improve interpretability. Autoscaling centered each variable by subtracting its mean and scaling by its standard deviation, ensuring equal contributions to the analysis regardless of their original scales

*SM2 Exploratory tools: Principal Component Analysis (PCA) and Data Reconstruction*

Following autoscaling, PCA was applied to reduce data dimensionality and visualize sample distributions. PCA is an unsupervised method for data reduction and visualization, decomposing the original data matrix into orthogonal variables known as Principal Components (PCs). These PCs capture the maximum variance in the data, defining a new space that highlights similarities and differences among samples.

In PCA, the coordinates of the data on the PCs, referred to as scores, represent the position of each sample within this new space. The loadings reflect the contribution of each original variable to the PCs, providing insight into the influence of individual variables on the PCs [57].

The PCA algorithm decomposes the original autoscaled data matrix ($X_{{mnc}_{\left( i,j \right)}}$). In general, it is possible to decompose the X_mnc matrix accordingly to Formula 1:

*Formula 1 PCA decomposition formula, where X_mnc is the 2-dimensional matrix of autoscaled data,, T are the pc scores values and P are the loadings and e is the remaining error matrix*

$$X_{{mnc}_{\left( i,j \right)}}=T_{\left( i,k \right)} P_{\left( k,j \right)}+E_{\left( i,j \right)}$$

Where *i* represents the number of samples and *j* the number of variables, into a set of orthogonal components:

where:

- $T_{\left( i,k \right)}$is the scores matrix representing the projections of the samples onto the PCs,
- $P_{\left( k,j \right)}$is the loadings matrix representing the contribution of each variable to the PCs,
- $E_{\left( i,j \right)}$is the residual matrix, capturing variance not explained by the PCs,
- *k* is the number of components.

To reduce experimental noise, initial PCA analyses used technical replicates and QC samples to identify PCs dominated by systematic artifacts, such as batch effects. PCs that primarily captured non-biological variance, such as batch effects or systematic artifacts, were identified based on visual inspection of score plots and variance explained by each PC. The dataset was reconstructed by retaining only the relevant PCs. Reconstruction was performed as follows:

$$X_{{mnc RECONSTRUCTED}_{\left( i,j \right)}}=T_{\left( i,kr \right)} P_{\left( kr,j \right)}$$

- *kr* is the number of retained components.

This process removed variability unrelated to biological differences while preserving the data's overall structure. The reconstructed data was used to calculate the mean of technical replicates, ensuring unbiased biological interpretations

The effectiveness of noise removal was assessed by comparing the variance explained before and after reconstruction.

*SM3 Chemometric classification models*

To classify the four distinct groups, we employed PLS-DA, a widely used supervised classification technique. This method involves applying PLS regression to a binary-coded response matrix (Y), where the predictor matrix (X) consists of independent spectra, and the class membership is represented in Y (e.g., [1 0 0 0] for the first class, [0 1 0 0] for the second class, and so on). The algorithm identifies the optimal relationship between X and Y to perform classification. The classification process is completed using Linear Discriminant Analysis (LDA) applied to either the predicted Y values or the latent variables. The number of Latent Variables (LVs) used in the model was optimized through a five-fold cross-validation procedure (Venetian blinds) to reduce misclassification errors and enhance accuracy [59,60].

Once the predictive models were constructed, their robustness and ability to generalize to new data were evaluated using repeated Double Cross-Validation (rDCV) [60]. This method was selected due to the limited sample size, ensuring an unbiased validation process [61]. Double cross-validation (DCV) involves two nested validation loops: the inner loop is responsible for model tuning, such as selecting the optimal number of LVs, while the outer loop assesses the model's predictive performance on unseen data (i.e., samples not used for training). The "repeated" nature of the method refers to multiple iterations of the process (50 repetitions in this study), with the data split differently in each iteration to minimize bias from any single partition. Additionally, to confirm that the model's performance was not due to chance, permutation tests were conducted, comparing the observed metrics with null distributions obtained through non-parametric methods [62].

Model performance was further evaluated using key metrics: sensitivity, specificity and accuracy. Together, these metrics offered a comprehensive evaluation of the model's classification performance [63]. In fact, sensitivity and specificity measure the percentage of True Positives (TP) and True Negatives (TN), respectively, and accuracy provides the overall proportion of correctly classified samples. In details True Positives (TP) represent the correctly identified samples of the target category, and True Negatives (TN) represent correctly identified non-target samples. False Positives (FP) indicate instances mistakenly classified as part of the target category, while False Negatives (FN) are those from the target category that were incorrectly classified.


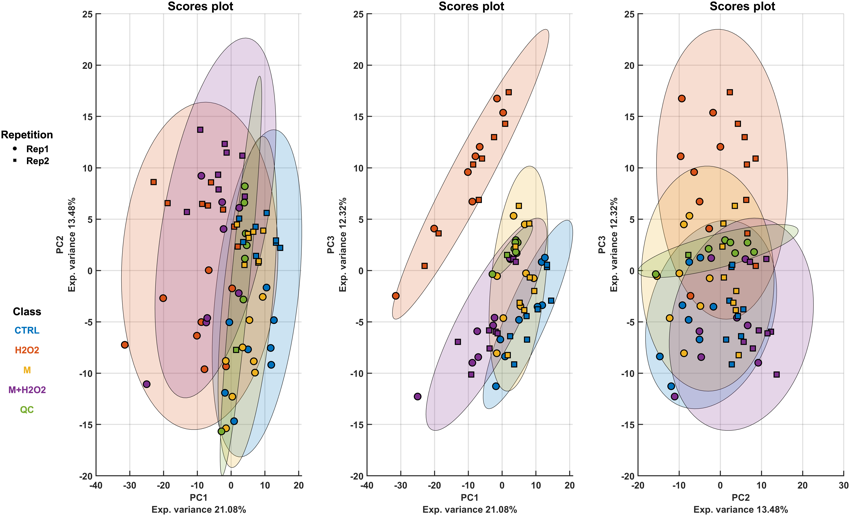

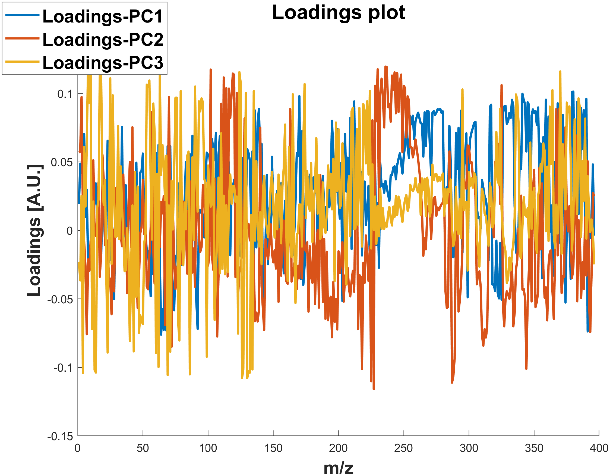


**Figure S2**: PCA scores and loadings plot. (A) PCA scores plot showing the distribution of samples along the first three principal components (PC1, PC2, PC3), which capture 21.08%, 13.48% and 12.32% of the total variance, respectively. The different clusters correspond to Control (CTRL), H_2_O_2_-induced oxidative stress in IEC-6 cells (H_2_O_2_), BLUBE in IEC-6 cells (M), H_2_O_2_-induced oxidative stress+BLUBE in IEC-6 cells (M+H_2_O_2_), and Quality Control (QC). Different replicates are indicated using distinct symbols (Replicate 1: circle, Replicate 2: square), showing clear separation between the replicates. Confidence ellipses (based on Hotelling's T² statistic for a 95% confidence interval) are overlaid to illustrate the variability within each group. These ellipses represent the spread of the data along the principal axes, showing the direction of maximum variance. (B) PCA loadings plot displaying the contribution of individual variables to PC1, PC2 and PC3. Variables with larger loadings are the most influential in driving the separation between the samples in the scores plot.


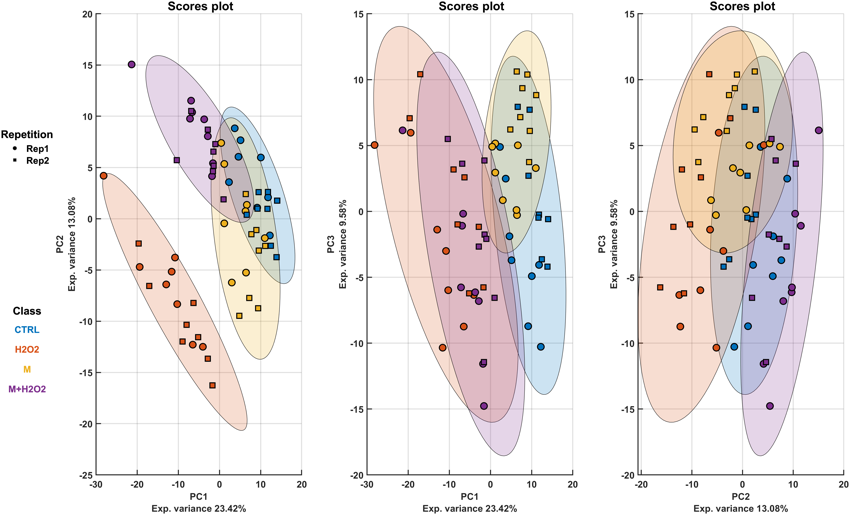

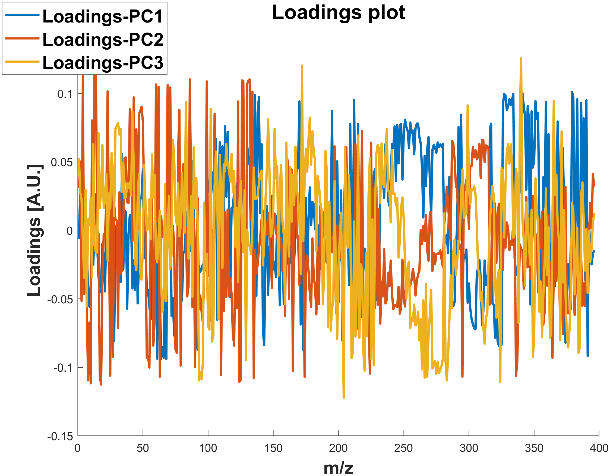


**Figure S3**: PCA scores and loadings plot after removing PC2 and reconstructing the data. (A) PCA scores plot showing the distribution of samples along the first three principal components (PC1, PC2, PC3), which capture 23.42%, 13.08% and 9.58% of the total variance, respectively. The different clusters correspond to Control (CTRL), H_2_O_2_-induced oxidative stress in IEC-6 cells (H_2_O_2_), BLUBE in IEC-6 cells (M), and H_2_O_2_-induced oxidative stress+BLUBE in IEC-6 cells (M+H_2_O_2_). Different replicates are indicated using distinct symbols (Replicate 1: circle, Replicate 2: square), showing minimized separations between the replicates. Confidence ellipses (based on Hotelling's T² statistic for a 95% confidence interval) are overlaid to illustrate the variability within each group. These ellipses represent the spread of the data along the principal axes, showing the direction of maximum variance. (B) PCA loadings plot displaying the contribution of individual variables to PC1, PC2 and PC3. Variables with larger loadings are the most influential in driving the separation between the samples in the scores plot.


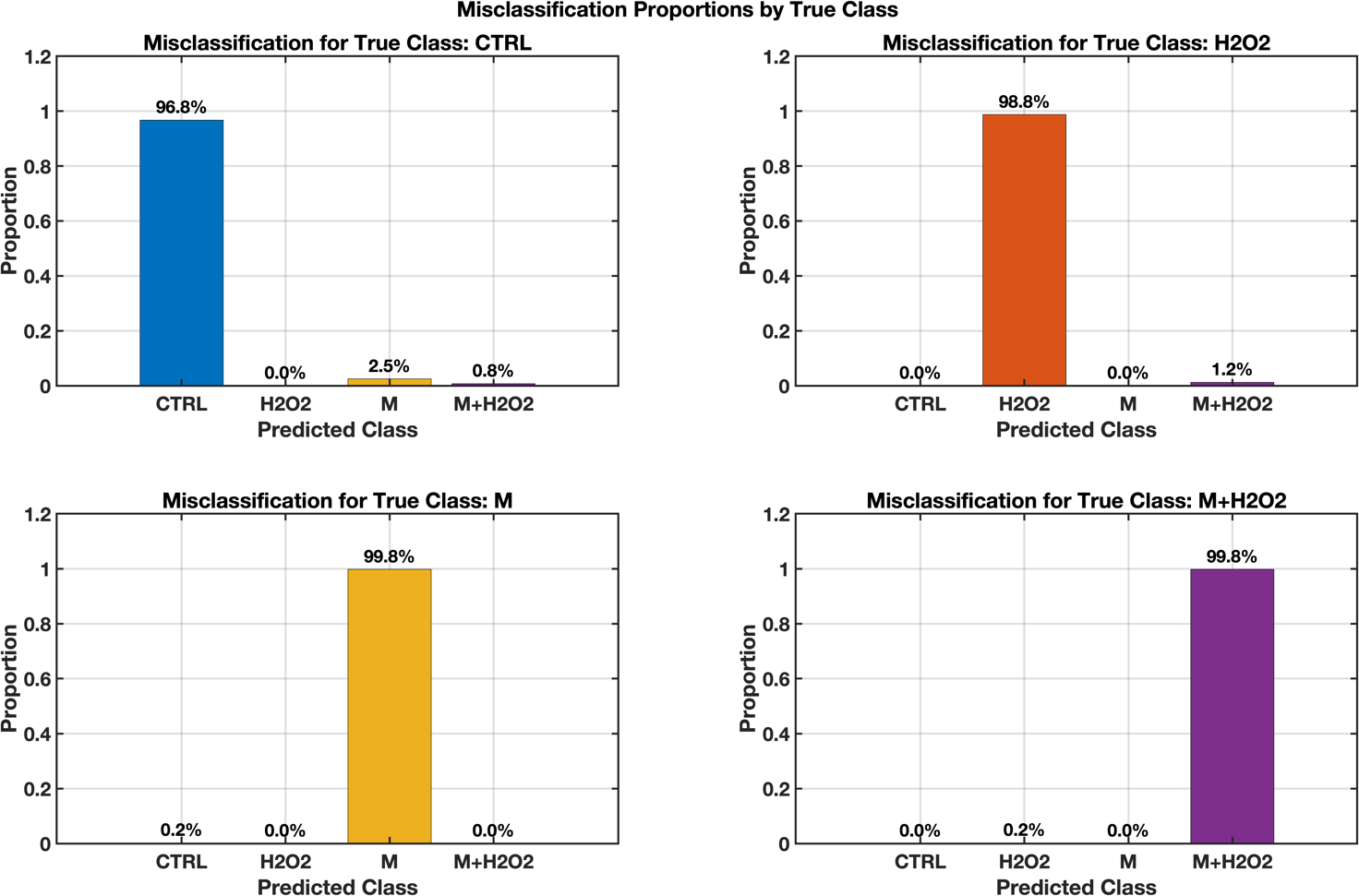


**Figure S4**. Misclassification Proportions for Each True Class in the Classification Model. Each subplot represents the classification performance for one of the four true classes: control (CTRL), H_2_O_2_-induced oxidative stress (H_2_O_2_), BLUBE treatment (M), and combined BLUBE- H_2_O_2_ treatment (M+ H_2_O_2_) in IEC-6 cells. The x-axis displays the predicted classes, while the y-axis shows the proportion of predictions. Bars are color-coded to represent the predicted classes, with the percentage of correct and misclassified instances labeled above each bar.


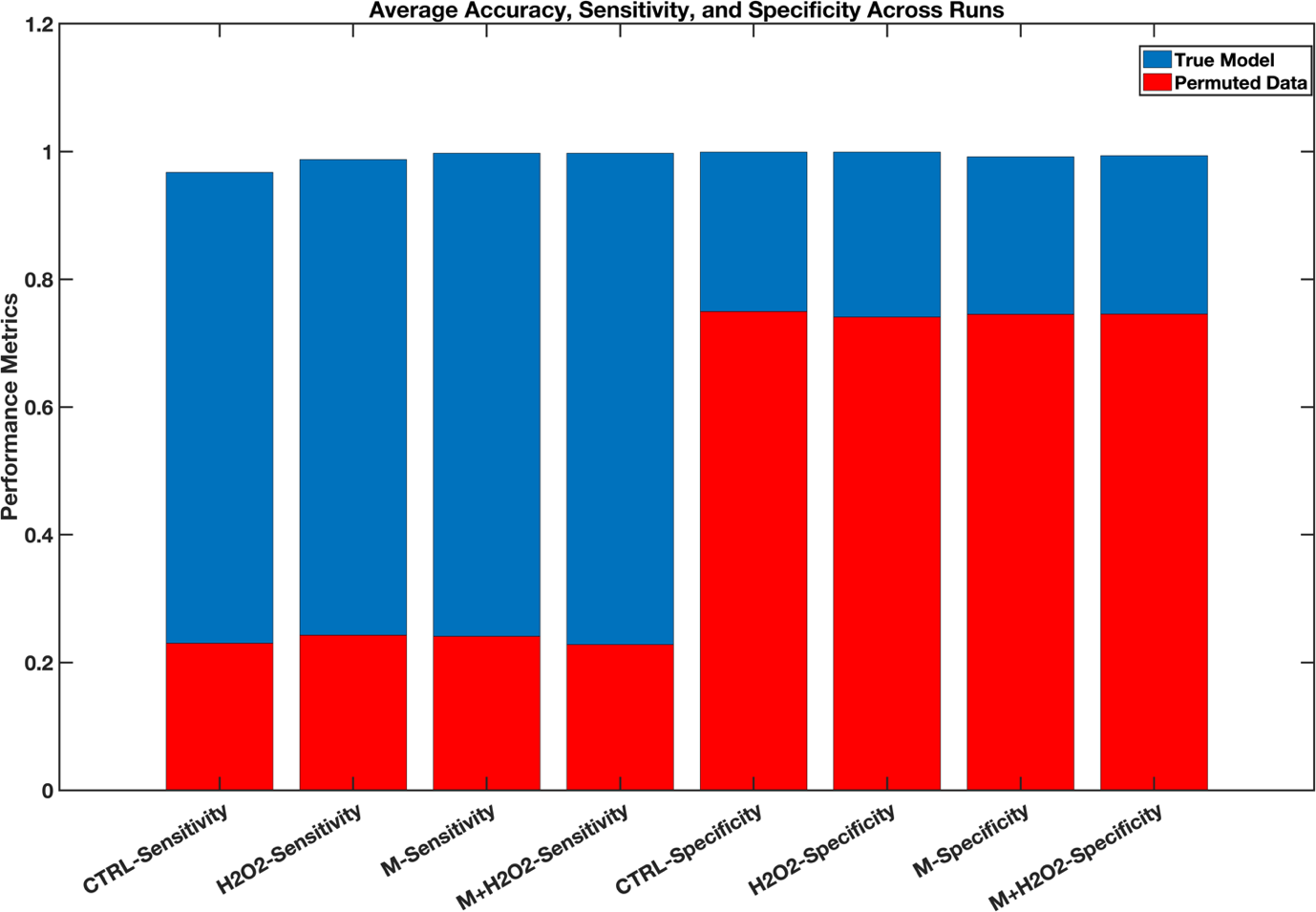


**Figure S5.** Performance metrics from rDCV and permutation tests using PLS-DA for the classification of four classes: control (CTRL), H_2_O_2_-induced oxidative stress (H_2_O_2_), BLUBE treatment (M), and combined BLUBE- H_2_O_2_ treatment (M+ H_2_O_2_) in IEC-6 cells. The rDCV was performed with 10 cancellation groups in both inner and outer loops, repeated 50 times. The blue bars represent the model's true sensitivity and specificity, while the red bars indicate the distribution of performance under 1000 permutations, serving as a null model.


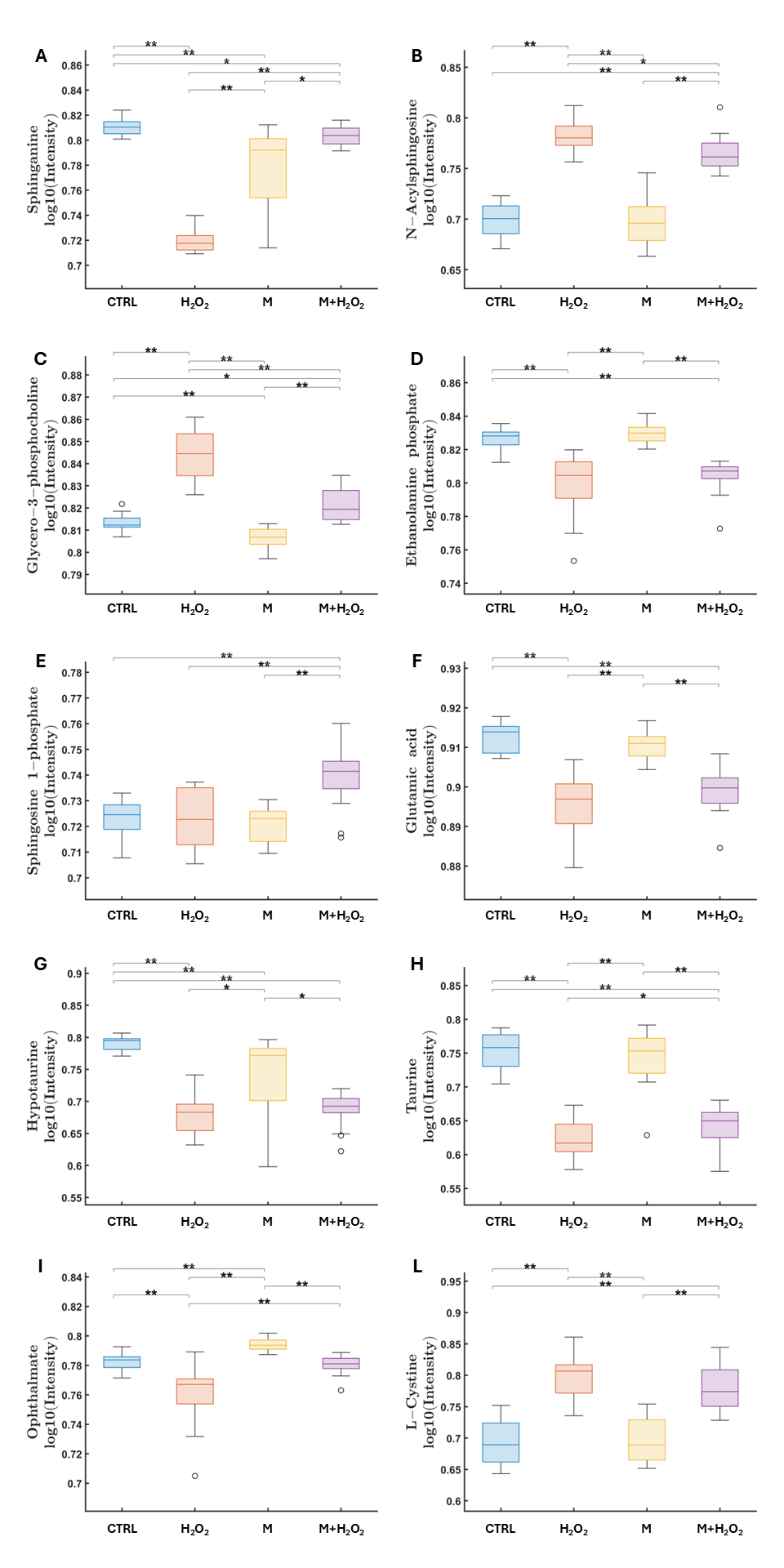


**Figure S6.** Distribution in Control (CTRL), H_2_O_2_-induced oxidative stress in IEC-6 cells (H_2_O_2_), BLUBE in IEC-6 cells (M), and H_2_O_2_-induced oxidative stress+BLUBE-IEC-6 cells (M+H_2_O_2_) of involved metabolites. **p-value* < 0.05 and ***p-value* < 0.01
